# Enhanced and Efficient Predictions of Dynamic Ionization through Constant-pH Adiabatic Free Energy Dynamics

**DOI:** 10.1021/acs.jctc.4c00704

**Published:** 2024-11-08

**Authors:** Richard S. Hong, Busayo D. Alagbe, Alessandra Mattei, Ahmad Y. Sheikh, Mark E. Tuckerman

**Affiliations:** † AbbVie Inc., Molecular Profiling and Drug Delivery, Research & Development, 1 N Waukegan Road, North Chicago, Illinois 60064, United States; ‡ Department of Chemistry, New York University, New York City, New York 10003, United States; § Courant Institute of Mathematical Sciences, New York University, New York, New York 10012, United States; ∥ NYU-ECNU Center for Computational Chemistry at NYU Shanghai, 3663 Zhongshan Road North, Shanghai 200062, China; ⊥ Simons Center for Computational Physical Chemistry at New York University, New York, New York 10003, United States

## Abstract

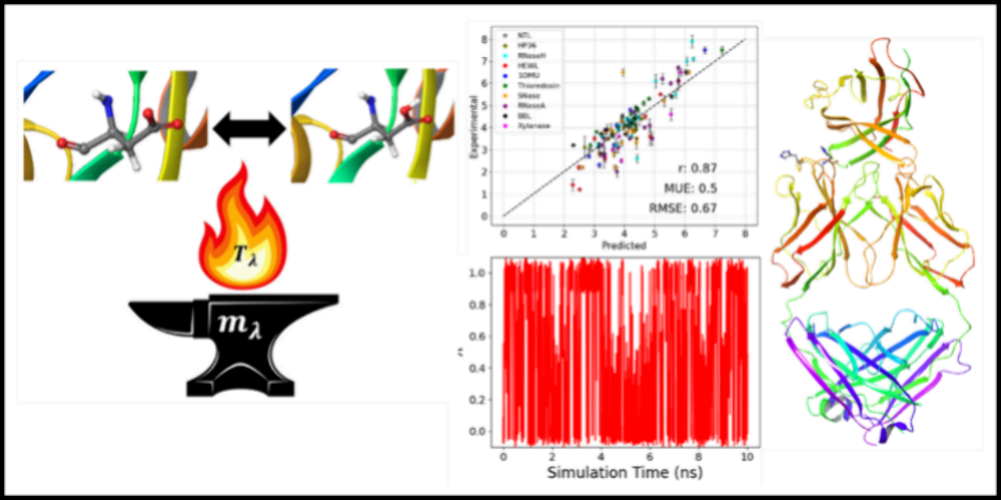

Dynamic or structurally induced ionization is a critical aspect of many physical, chemical, and biological processes. Molecular dynamics (MD) based simulation approaches, specifically constant pH MD methods, have been developed to simulate ionization states of molecules or proteins under experimentally or physiologically relevant conditions. While such approaches are now widely utilized to predict ionization sites of macromolecules or to study physical or biological phenomena, they are often computationally expensive and require long simulation times to converge. In this article, using the principles of adiabatic free energy dynamics, we introduce an efficient technique for performing constant pH MD simulations within the framework of the adiabatic free energy dynamics (AFED) approach. We call the new approach pH-AFED. We show that pH-AFED provides highly accurate predictions of protein residue p*K*_a_ values, with a MUE of 0.5 p*K*_a_ units when coupled with driven adiabatic free energy dynamics (d-AFED), while reducing the required simulation times by more than an order of magnitude. In addition, pH-AFED can be easily integrated into most constant pH MD codes or implementations and flexibly adapted to work in conjunction with enhanced sampling algorithms that target collective variables. We demonstrate that our approaches, with both pH-AFED standalone as well as pH-AFED combined with collective variable based enhanced sampling, provide promising predictive accuracy, with a MUE of 0.6 and 0.5 p*K*_a_ units respectively, on a diverse range of proteins and enzymes, ranging up to 186 residues and 21 titratable sites. Lastly, we demonstrate how this approach can be utilized to understand the in vivo performance engineered antibodies for immunotherapy.

## Introduction

The ionization states of molecules play a critical role in numerous biochemical, biological, and physicochemical processes, and are commonly characterized by their acid ionization constants or p*K*_a_ values. These ionization states are not solely determined by the intrinsic properties of a molecule or a particular protein residue but are also influenced by the dynamic chemical environment and the intricate conformational landscape of a system. In a study by Onufriev et al., for example, the authors demonstrated that in over 60% of small molecule-protein interactions, at least one ionizable residue in the protein exhibited a different ionization state between its free and bound states.^1^ Small molecule drugs can dynamically shift their ionization or tautomeric states at physiologically relevant pH conditions to enable crucial biological and biophysical processes, such as membrane permeation, recrystallization, salt disproportionation, and binding to biological targets.^2−6^ In addition, by leveraging the environment-dependent protonation states of histidine residues, pH-selective monoclonal antibodies can be designed to specifically target the acidic tumor environments, thereby preventing dose-limiting toxicity.^7^ Lastly, the ionization states of a macromolecule define its isoelectric pH value, which can have significant implications for its physicochemical and formulation properties.^8,9^ For these reasons, it becomes essential to accurately capture the ionization states of both small molecules and protein residues across diverse chemical environments.

Experimental techniques, including NMR titration, can be used to determine p*K*_a_ values and ionization or tautomeric states for large biomolecules, but they can be resource-intensive and challenging to conduct. Furthermore, identifying ionization states throughout dynamic processes, such as membrane permeation, or in dynamic systems, such as intrinsically disordered proteins, can be even more complex. Given these challenges, computational techniques have become essential for predicting both the static and dynamic ionization states of complex biomolecular structures or biophysical systems. From these computational approaches, empirical and data-driven schemes, such as protein pKa (PROPKA), have been widely used to quickly assign ionization states for protein residues from X-ray structures.^10,11^ In recent years, neural network approaches based on deep representation learning have also been developed for rapid and precise determination of protein p*K*_a_ values.^12,13^ However, since these methods only account for the static environment around each ionizable residue and do not consider the effect of conformational dynamics on the ionization states, they may lose predictive power in disordered structures or dynamic systems. To address this limitation, constant pH molecular dynamics (pHMD) methodologies have been developed to predict and study the ionization states and p*K*_a_ values in systems subject to fluctuating chemical environments.^14−16^

Constant pH molecular dynamics, as first introduced by Baptista et al., enables the ionization states of each titratable site during a molecular dynamics (MD) simulation to change dynamically based on the surrounding chemical environment and electrostatics of each titratable site.^15,17^ This implementation, known as discrete constant pH MD, involves sampling the ionization sites using discrete Monte Carlo steps throughout the MD simulation, where the energetics of protonation are described using Poisson–Boltzmann (PB) electrostatics.^18^ For systems involving hydrophobic pockets^19,20^ and trans-membrane channels,^21,22^ explicit consideration of water molecules is essential to accurately capture the system’s behavior. Free Energy Perturbation (FEP) techniques have recently been utilized to predict precise single residue p*K*_a_ values in explicit solvent.^23^ However, these approaches encounter challenges due to the exponentially increasing computational costs for residues with multiple titratable sites and the difficulty in capturing tightly coupled ionization states for certain residues.^24^ To address these limitations, a continuous approach based on the λ dynamics method by Khandogin et al. can be utilized.^14^ In this approach, the ionization state of each residue is described by a one-dimensional λ coordinate, λ ∈ [0,1], that can dynamically fluctuate throughout the MD simulation. To promote the sampling of physically relevant end-point states, a barrier potential, as outlined by Rosso et al. and Lee et al., can be introduced between λ = 0 and λ = 1, along with an enhanced sampling algorithm to ensure efficient crossing of the barrier.^14,25^ Since its initial development, the continuous λ/constant pH molecular dynamics (CpHMD) approach has undergone further advancements and has been implemented in various open-source MD packages including AMBER, CHARMM, and GROMACS.^14,16,20,26−28^ These implementations have been designed to be scalable, user-friendly, computationally efficient, and compatible with graphics processing units (GPUs), allowing for accelerated simulations.^27,28^

In addition, the CpHMD approach has been adapted for all-atom explicit solvent simulations, enabling more accurate predictions and descriptions of complex systems where water molecules play a crucial role.^26,28−31^ However, existing CpHMD approaches, notably for explicit solvent simulations, face challenges in achieving rapid convergence of p*K*_a_ values, especially for deeply buried residues or those involved in salt bridges. These challenges often require significantly extended simulation times to accurately capture the ionization behavior of these residues.

To address the challenge of poor sampling, multiple research groups have developed different versions of enhanced constant pH MD. One popular approach for enhancing the sampling of CpHMD simulations, first introduced by Wallace and Shen, is the use of pH-replica exchange methods.^20,32−34^ These methods involve running multiple simulations at different pH values in parallel and exchanging either the protonation λ coordinate or the simulation coordinates between adjacent replicas. Temperature-based replica exchange methods, such as parallel tempering, have also been employed to enhance the sampling of molecular configurations in CpHMD simulations.^35,36^ The requirement of multiple closely spaced replicas to ensure sufficient overlap for replica exchange between different states results in significant computational overhead. Furthermore, due to the requirement of multiple parallel replicas, it is often not straightforward to augment this approach with other enhanced MD approaches that bias the molecular coordinates.

In this study, we introduce the pH-AFED (Adiabatic Free Energy Dynamics) method, a new approach to enhance the sampling of the multidimensional titration **λ**-coordinate space in CpHMD simulations. Like the adiabatic free energy dynamics (AFED) framework described by Rosso et al.,^25^ we maintain the temperature of the **λ** coordinates at a high value *T*_λ_, while adiabatically decoupling them from the physical system through a large fictitious mass assignment *m*_λ_. This enables rapid sampling of the **λ** coordinates, facilitating efficient and more accurate predictions of residue p*K*_a_ values in all-atom explicit solvent CpHMD simulations without the requirement of multiple parallel replicas nor swapping of configurations between different simulations. As such, simulations at each independent pH can be easily conducted on a single GPU and paired with other enhanced sampling approaches that drive the molecular coordinates. Our results demonstrate that pH-AFED achieves rapid sampling of the multiple titration coordinates in systems that are typically challenging for standard all-atom CpHMD simulations in explicit solvents. Because of short simulation times, more efficient and practical predictions of ionization states can be also applied to complex systems.

To validate the pH-AFED approach, we compared the performance of pH-AFED with standard GROMACS all-atom explicit solvent CpHMD on larger biological systems, including the well-studied hen egg white lysozyme (HEWL, PDB ID: 2LZT), as well as a diverse set of proteins and enzymes, with up to 186 residues and 21 titratable sites. We show that by effectively sampling the **λ** coordinates, pH-AFED significantly enhances the sampling of ionization states, leading to more accurate predictions of residue p*K*_a_ values than can be achieved in standard GROMACS all-atom CpHMD simulations. Next, we demonstrate how pH-AFED can be combined with other enhanced sampling techniques to improve the sampling of molecular conformations, resulting in more accurate predictions with shorter simulation times. Lastly, we show how this ensemble enhanced sampling approach with pH-AFED and enhanced sampling of the molecular coordinates can be utilized to effectively predict ionization states of large monoclonal antibody complexes to enable rapid selective antibody design.

## CpHMD Methodology

In this study, we utilize the CpHMD scheme in GROMACS, as recently implemented by Aho et al., which uses a charge interpolation scheme for computing electrostatics.^28^ Similar to the lambda dynamics formulation, the Hamiltonian is prescribed in eq 1.
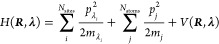
1Here, *V*(***R***,**λ**) denotes the potential energy function as a function of both the Cartesian coordinates and the complete set of titration coordinates **λ**. The potential energy function, *V*(***R***,λ) of a single titratable site is given in eq 2, where states A and B describe the two different possible protonation states.
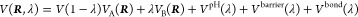
2

The terms *V*_A_(***R***) and *V*_B_(***R***) represent the potential energy function, as described by the partial charges of ionization state A or state B. The *V*^pH^(λ) term in eq 2 denotes the pH-dependent potential, where a step function potential is utilized. Further details of the equations used for *V*^pH^(λ) and *V*^barrier^(λ) are described in the Supporting Information (ESI).

### Multiple Titration States and Charge Constraints

For multiple ionization sites, the potential energy function, *V*(***R***,**λ**), is determined as the summation of the contributions from eq 2, , for all titratable residues. In the CpHMD simulations performed in this study, charge neutrality is maintained at each step using multiple coupled titratable buffer particles, as described by Donnini et al., where a “SHAKE”-like constraint is applied at the end of each **λ** time step. For multiple titratable sites, there will be a constraint equation, described by eq 3, to maintain charge neutrality.
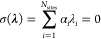
3Here, α_*i*_ is defined as *N*_p_(*q*_A_–*q*_B_) where *N*_p_ is the number of coupled sites for each titratable λ, and *q*_A_ and *q*_B_ represent the total partial charges of each protonation state. *N*_p_ will typically be 1 for relevant titratable residues and greater than 1 for the coupled titratable buffer particles. Eq 3 leads to a coupling between the *N*_sites_ λ degrees of freedom.

Next, a Lagrange multiplier ζ, which is determined by solving eq 4, can be used to correct each of the unrestrained **λ** values, λ_*i*_^u^, through eq 5.
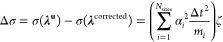
4
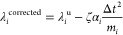
5

Further details regarding the derivations of the constraint equations and the application of these constraints along with multisite residues in histidine can be found in the appendix of ref (28).

### pH-AFED Methodology

In standard λ dynamics and CpHMD simulations, the temperature of the dynamic λ variable(s) is set to match the simulation temperature while the mass is set to an arbitrary value that ensures sufficient sampling and adequate residence times at the end points λ = 0 or λ = 1. However, in the pH-AFED scheme, we assign a large fictitious temperature *T*_λ_ to the **λ** variables, allowing for rapid barrier crossing.^25,37^ Additionally, we assign a large fictitious mass, *m*_λ_, to decouple the dynamics of the **λ** variables from that of the physical system. Here, the large *m*_λ_ allows the λ variable to evolve on a slower time scale compared to coordinates of the physical system. Through this mechanism, the λ variables are able to sample the probability distribution *P*(**λ**) on the fly as the simulation proceeds. The high temperature *T*_λ_ increases the efficiency of sampling such that the free energy can be recovered via eq 6 below.

For higher *T*_λ_ values, we increase *V*_barrier_, the barrier between λ_*i*_ = 0 or λ_*i*_ = 1, to ensure better sampling at the physically relevant end points. The free energy profile for the **λ** variables can be determined using eq 6. Since adjacent titratable residues may be potentially coupled and are reweighted through eq 6, it is important to set adjacent λ particles at the same *T*_λ_.
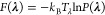
6

For a more detailed derivation of the equations of motion and of eq 6, readers can refer to the ESI and refs (25 and 37).

In contrast to many standard CpHMD approaches, which use a global thermostat to control the average temperature of all **λ** variables, pH-AFED implements independent thermostats for each independent λ_*i*_ variable (also known as “massive” thermostatting). This is necessary to maintain a constant high *T*_λ_ for each titratable residue and prevent the occurrence of “hot” and “cold” zones that can result from global thermostats in systems with multiple titratable sites. The presence of such zones can lead to either inadequate sampling of “cold” **λ** variables, or nonadiabaticity of “hot” **λ** variables, when the *T*_λ_ becomes too high for its respective *m*_λ_. By using individual thermostats in pH-AFED, we ensure that each λ_*i*_ variable maintains its desired temperature and avoids undesired temperature variations that can negatively impact the sampling and accuracy of the simulations.

Due to the requirements of maintaining charge neutrality through a “SHAKE” like charge constraint at the end of each **λ** integration step, independent thermostats inadvertently will result in an incorrect distribution of the momenta for each λ_*i*_ variable. To mitigate this issue, a thermostat algorithm using the velocity-verlet “side” scheme, is utilized for the λ_*i*_ momenta, *p*_λ_*i*__ such that a half thermostat step is incorporated before the **λ** coordinates and momenta are propagated.^38^ In addition, *n* multiple titratable buffer particles, as described by Donini et al.,^39^ are used for each titratable residue to minimize the impact of the charge constraint step on the momenta of the titratable **λ** variables. Further details and justification are described in the ESI. While we acknowledge that these mitigation schemes still do not allow for precise temperature control of *T*_λ_, they do enable approximate control of the temperature, which is sufficient to enable facile barrier crossing in **λ** space.

To enable histidine tautomerization in the pH-AFED scheme, a similar strategy is employed as in previous implementations where histidine tautomerization is described through a multisite representation.^28^ Here, a separate λ coordinate is assigned to each possible histidine tautomer *k*, where λ = 0 corresponds an unphysical reference state. Each λ coordinate here is parametrized using the respective microscopic p*K*_a_ values of the corresponding tautomeric states. In the standard GROMACS CpHMD approach, the constraint  is enforced at each step, representing the sum of the **λ** coordinates for the three distinct protonation or tautomeric states *k* for each histidine residue *i*. This constraint ensures sampling of physically relevant states throughout the simulation. We acknowledge that a more direct description can also be implemented, as formulated by Khandogin and Brooks, where each of the equilibria are directly modeled and coupled to the protonated end point.^16^

In pH-AFED, the use of massive or independent thermostats makes it challenging to incorporate multisite constraints. To overcome this issue, a hyperbolic tangent restraining potential, as described in eq 7, is introduced to maintain the sum of the tautomer λ states close to 1.00. This effectively captures the physically relevant tautomeric states.
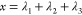

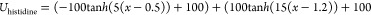
7

## Results and Discussion

### Validation on HEWL

We first tested our approach on the well benchmarked hen egg white lysozyme (HEWL) system, which consists of 10 titratable residues, using both pH-AFED and the standard GROMACS CpHMD approach. Here, 10 ns titration simulations were performed from pH = 0 to pH = 9 in intervals of 1.0 for pH-AFED and both 10 and 50 ns for standard GROMACS CpHMD. From only 10 ns titration simulations using pH-AFED with *T*_λ_ = 750 K, *m*_λ_ = 750, *V*_barrier_ = 10 kJ/mol, we observed improved agreement with the experimental values (MUE of 0.60 and RMSE of 0.68) compared to that from both 10 and 50 ns of titration simulations from the standard GROMACS CpHMD approach (Table 1). In addition, we demonstrate that for different *T*_λ_, *m*_λ_, and *V*_barrier_ combinations, we obtain similar prediction accuracies (ESI).

**Table 1:** pK_a_ Prediction Results from 10 ns of pH-AFED and Standard GROMACS CpHMD as well as 50 ns of Standard CpHMD^a^

residue	pH-AFED (10 ns)	standard GROMACS CpHMD (10 ns)	standard GROMACS CpHMD (50 ns)	exp.
Glu-7	3.27 ± 0.06	2.60 ± 0.16	2.64 ± 0.07	2.60
His-15	5.14 ± 0.04	4.95 ± 0.14	4.68 ± 0.00	5.50
Asp-18	3.13 ± 0.10	3.07 ± 0.35	3.42 ± 0.15	2.80
Glu-35	6.49 ± 0.27	8.56 ± 0.50	8.01 ± 0.89	6.10
Asp-48	2.07 ± 0.47	0.37 ± 0.67	1.35 ± 0.53	1.40
Asp-52	5.03 ± 0.02	5.73 ± 0.51	5.62 ± 0.00	3.60
Asp-66	1.96 ± 0.07	–0.08 ± 0.66	1.52 ± 0.01	1.20
Asp-87	2.75 ± 0.03	2.14 ± 0.36	1.96 ± 0.07	2.20
Asp-101	4.90 ± 0.11	5.37 ± 0.36	5.34 ± 0.00	4.50
Asp-119	3.01 ± 0.07	2.57 ± 0.37	2.94 ± 0.17	3.50
**MUE**	0.60	0.96	0.74	
**RMSE**	0.68	1.24	1.00	

a Error bars are derived from standard deviations of three independent simulations.

**Figure 1:**
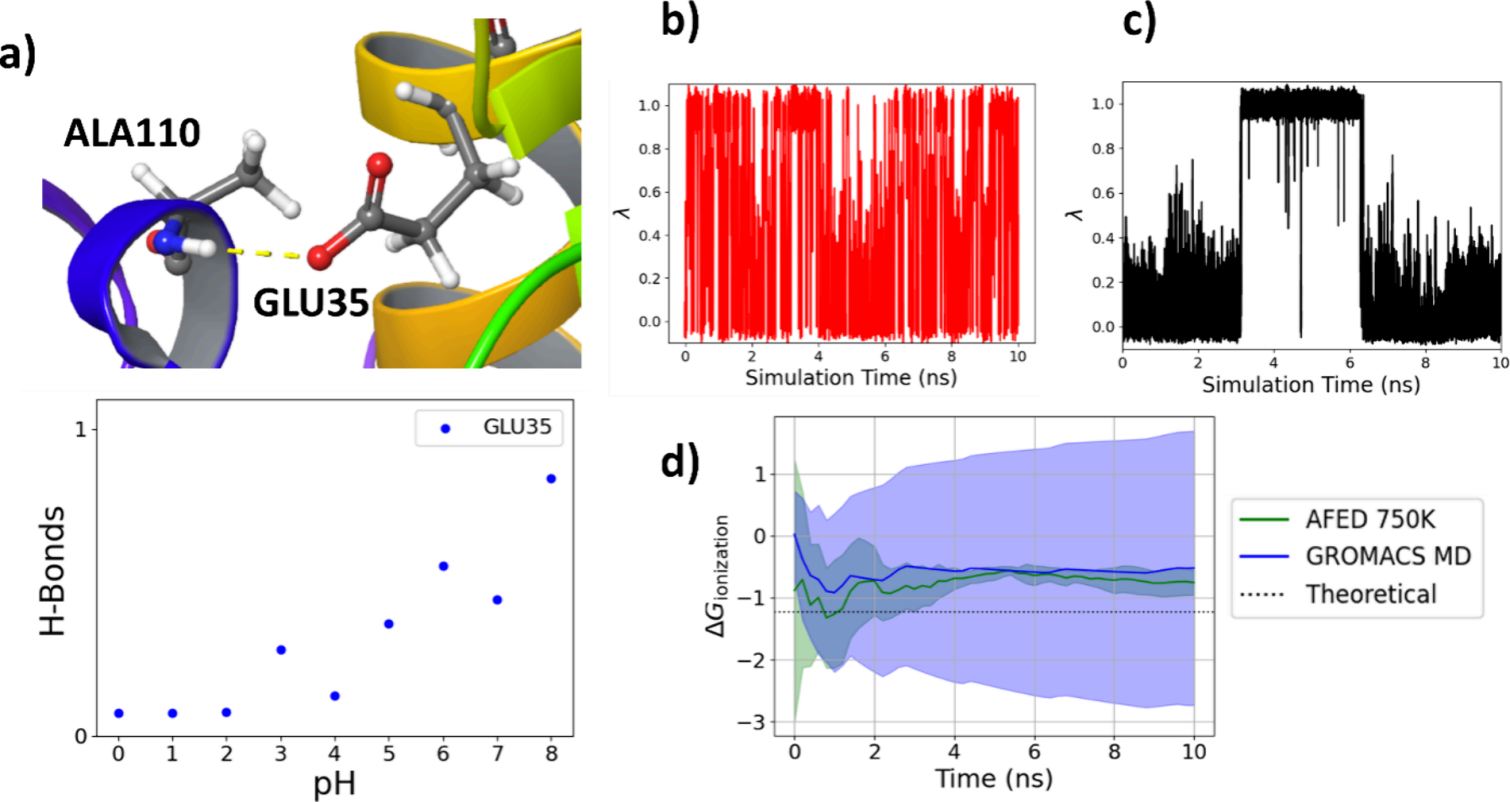
(a) Glu-35 residue of HEWL showing hydrogen bonding involving the ionized carboxylic acid and pH-dependent hydrogen bonding of Glu-35. (b) λ vs time for pH-AFED at pH 7. (c) λ vs time for the standard GROMACS CpHMD simulation at pH 7. (d) Ionization energy vs time for pH-AFED and standard GROMACS CpHMD at pH 7, where the average and standard deviation between three different simulations is shown in the green shading. Here, the ionization energy is computed as  using the cumulative protonated and deprotonated fractions. Standard GROMACS CpHMD, which had nonfinite ionization energies, were not used.

For the partially buried residue of Glu-35, which exhibits a solvent accessible surface area (SASA) of 27 Å^2^ and an ionized carboxylic acid that is involved in hydrogen bonding, pH-AFED demonstrated rapid sampling of the ionization energies, and improved convergence compared to the standard GROMACS CpHMD (Figures S1 and S2 in the ESI). In contrast, the standard GROMACS CpHMD approach faced challenges in sampling, as depicted in Figure 1b,d, where significant variations in ionization energy were observed between independent simulations. From pH-AFED, we also observed similar pH-dependent hydrogen bonding of Glu-35 to that reported by Harris et al.^26^ However, notable deviations from the experimental results were observed for Asp-52 in all approaches. This discrepancy may be attributed to the inability of the force field to capture accurately the interactions of this residue.^28^

### Validation of pH-AFED on the Diverse Protein Set

Next, we performed a larger scale validation of the pH-AFED approach on a diverse set of proteins, enzymes, and RNAs, compiled from various literature sources,^27,40−44^ and compared the performance to that of standard GROMACS CpHMD. Here, we have set the parameters to *T*_λ_ = 750 K, *m*_λ_ = 750, *V*_barrier_ = 10 kJ/mol to use for larger systems for most residues, where residues that exhibit a Solvent Accessible Surface Area (SASA) of less than 10 Å^2^ will use a *T*_λ_ = 1500 K, *m*_λ_ = 1500, *V*_barrier_ = 22 kJ/mol. Titration simulations were performed from pH = 0 to pH = 9 in intervals of 1, where each independent simulation was run for 10 ns.

From testing pH-AFED on this extended set of proteins, we observed, overall, reasonable agreement with the experimental p*K*_a_ values with a mean unsigned error (MUE) and root-mean-square-error (RMSE) of 0.60 and 0.80, respectively, showing significant improvement compared to simulations from 10 ns of the standard GROMACS CpHMD approach, which exhibits an overall MUE and RMSE of 0.84 and 1.27, respectively. From these results, we demonstrate that pH-AFED can precisely and efficiently predict residue p*K*_a_ values of proteins that have more than 20 titratable residues.

**Figure 2:**
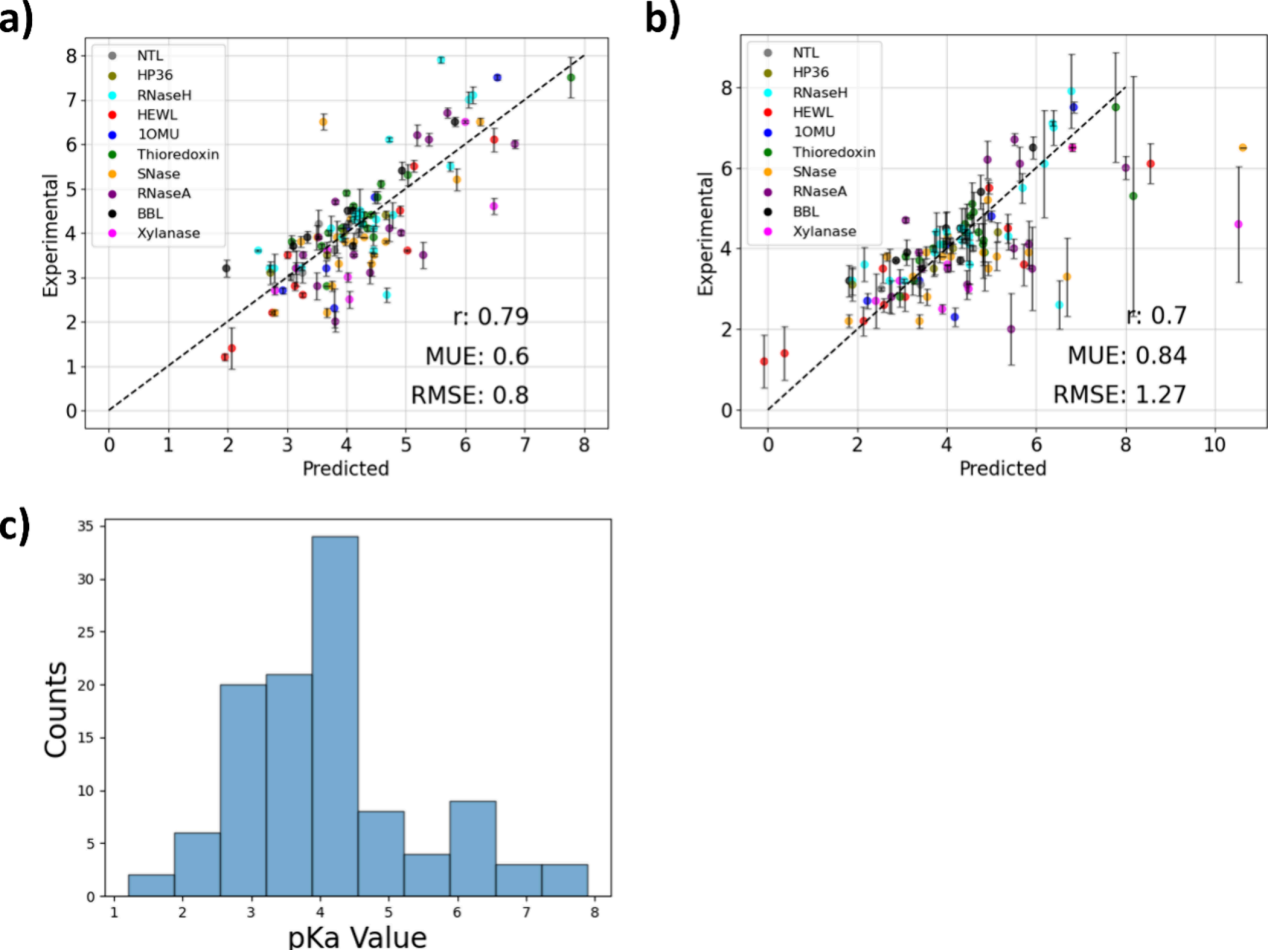
(a) Prediction results from pH-AFED for various proteins from 10 ns of pH-AFED simulations. (b) Prediction results for various proteins from 10 ns of standard GROMACS CpHMD simulations. Mean unsigned error (MUE), root mean squared error (RMSE), and Pearson correlation coefficient (*r*) are reported for each set. (c) Spread of p*K*_a_ values from the validation set used in this study.

**Figure 3:**
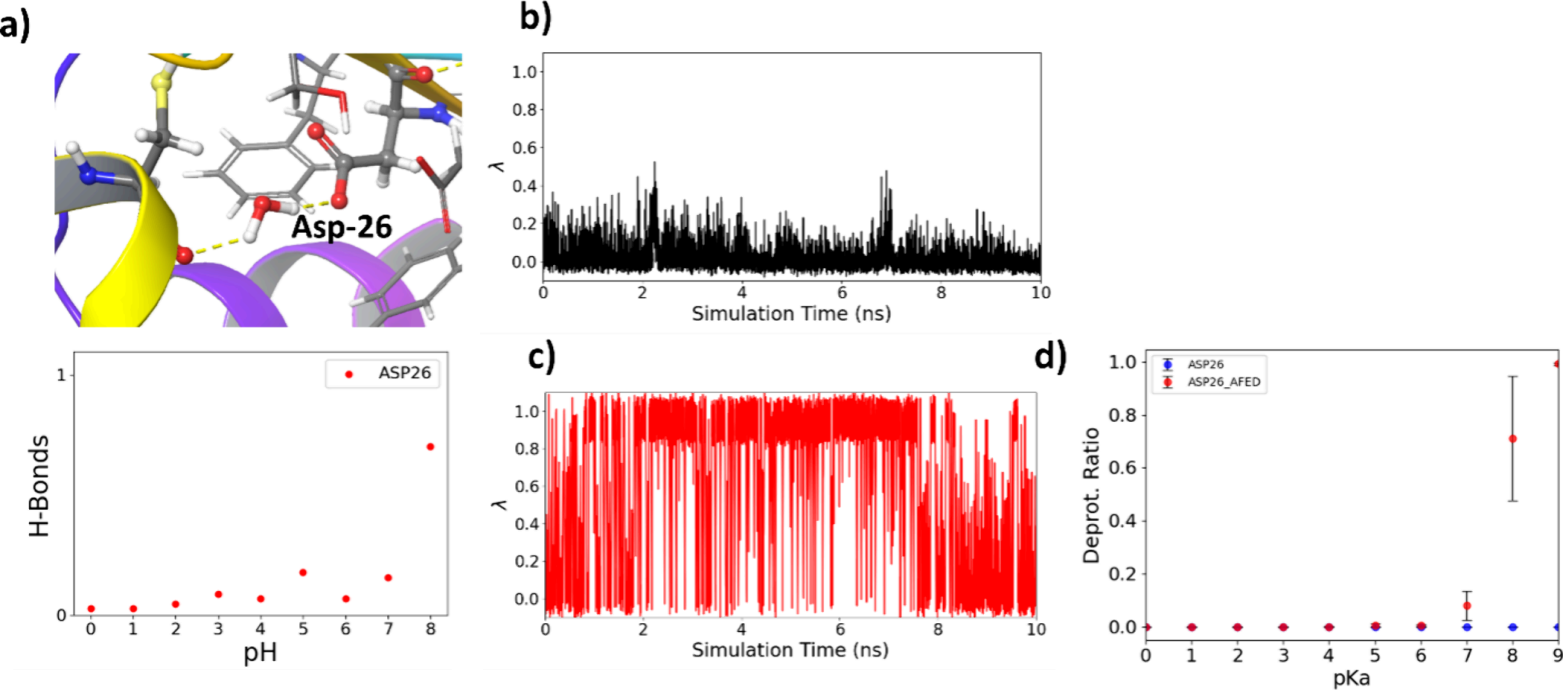
(a) Image depicting the buried chemical environment of Asp-26 (shown in pink) in the Thioredoxin protein. (b) 100 ns standard GROMACS CpHMD λ trajectory of Asp-26 at pH 8. (c) 10 ns pH-AFED λ trajectory of Asp-26 at pH 8. (d) Titration curve for standard GROMACS CpHMD (blue) and for pH-AFED (red) where error bars are computed through the standard deviation of three different simulations.

The tabulated results from both methods are shown in the ESI. We also observed that pH-AFED achieves significantly improved sampling compared to the standard GROMACS CpHMD approach for highly buried residues (Figure 2) such as the Asp-26 residue of the Thioredoxin protein, a highly buried residue which exhibits a SASA of 0 Å^2^. Here, with the standard GROMACS CpHMD approach, a titration curve cannot be generated due to poor sampling of the λ coordinate in the simulations, even up to a simulation length of 100 ns (Figure 3). This could possibly be attributed to the highly stabilized ionization or λ state of the starting configuration through extensive hydrogen bonding. However, by using pH-AFED, we can achieve rapid sampling of the ionization states of Asp-26, leading to physically relevant predicted titration curves (Figure 3). In addition, from pH-AFED, we observed similar pH-dependent intraprotein hydrogen bonding and salt bridge formation trends of Asp-26 as those mentioned by Harris et al.^26^

While the p*K*_a_ predictions for the highly buried residue of Asp-26 closely matched that of the experimental value of 7.5,^45^ we observed other buried residues such as Asp-21 of SNase, Asp-14 of RNaseA, and Glu-79 of Xylanase were present as notable outliers with errors of 2.9, 1.81, and 1.88 respectively. While the high *T*_λ_ used for buried residues allowed rapid sampling of the λ coordinate, we suspect that slow conformational sampling can contribute to these discrepancies. Trajectory analysis of key torsion angles of these residues showed, indeed, slow conformational sampling (Figure S3). In addition, the presence of mixed states, as characterized by **λ** values between 0.2 and 0.8, may affect the prediction of p*K*_a_ values and the system dynamics. While mixed states are frequently sampled in the pH-AFED trajectory, as observed in Figures 1 and 3, their overall free energy contributions are reduced due to the presence of the barrier potential and the weighting of the probability distributions using eq 6 (further details are described in the ESI). After reweighting the mixed fraction contributions, we still, however, observe a large percentage of mixed fractions for many residues using the pH-AFED simulation parameters outlined earlier (Table S5). We show (Tables S5 and S6) that simulations using a larger barrier potential can reduce the fraction of mixed states while maintaining the same predictive accuracy.

Since pH-AFED does not require simulations involving multiple parallel replicas nor the need to exchange configurations between replicas, seamless integration of pH-AFED with other enhanced sampling methods that bias the molecular coordinates can be applied. Here, in order to alleviate the challenge of slow conformational sampling, we incorporated enhanced sampling of relevant torsions for the titratable residues through the driven adiabatic free energy dynamics (d-AFED) approach.^46^ While the CHARMM36 force field that was used in this study, as described by Buslaev et al., incorporates torsional modifications with a smaller torsional barrier, additional enhancements are still required to rapidly sample torsion angles for buried and semiburied residues. The following torsion angles were used as collective variables for Asp Residues: N–C_γ_–C_β_–C_α_, O_α_–C_δ_–C_α_–C_β_, and H–O_δ2_–C_δ_–O_δ1_ torsion angles; for Glu Residues: N–C_γ_–C_β_–C_α_, C_δ_–C_α_–C_β_–C_γ_, O_δ1_–C_δ_–C_α_–C_β_, and H–O_δ2_–C_δ_–O_δ1_ torsion angles; for His Residues: N–C_α_–C_β_–C_γ_, and H–N–C_α_–C_β_. Depictions of these torsion angles are shown in Figure S1 of the ESI. Note that very few collective-variable (CV) based sampling methods can handle many target CVs. Here, d-AFED was used to enhance the sampling of these torsion angles for titratable residues that exhibit a SASA of less than 80 Å^2^. Similar to the AFED approach, d-AFED allows efficient sampling of a large number of collective variables, conducting full sweeps of multidimensional free energy landscapes.^46^ Further details about the d-AFED simulations are described in the computational details section.

**Figure 4:**
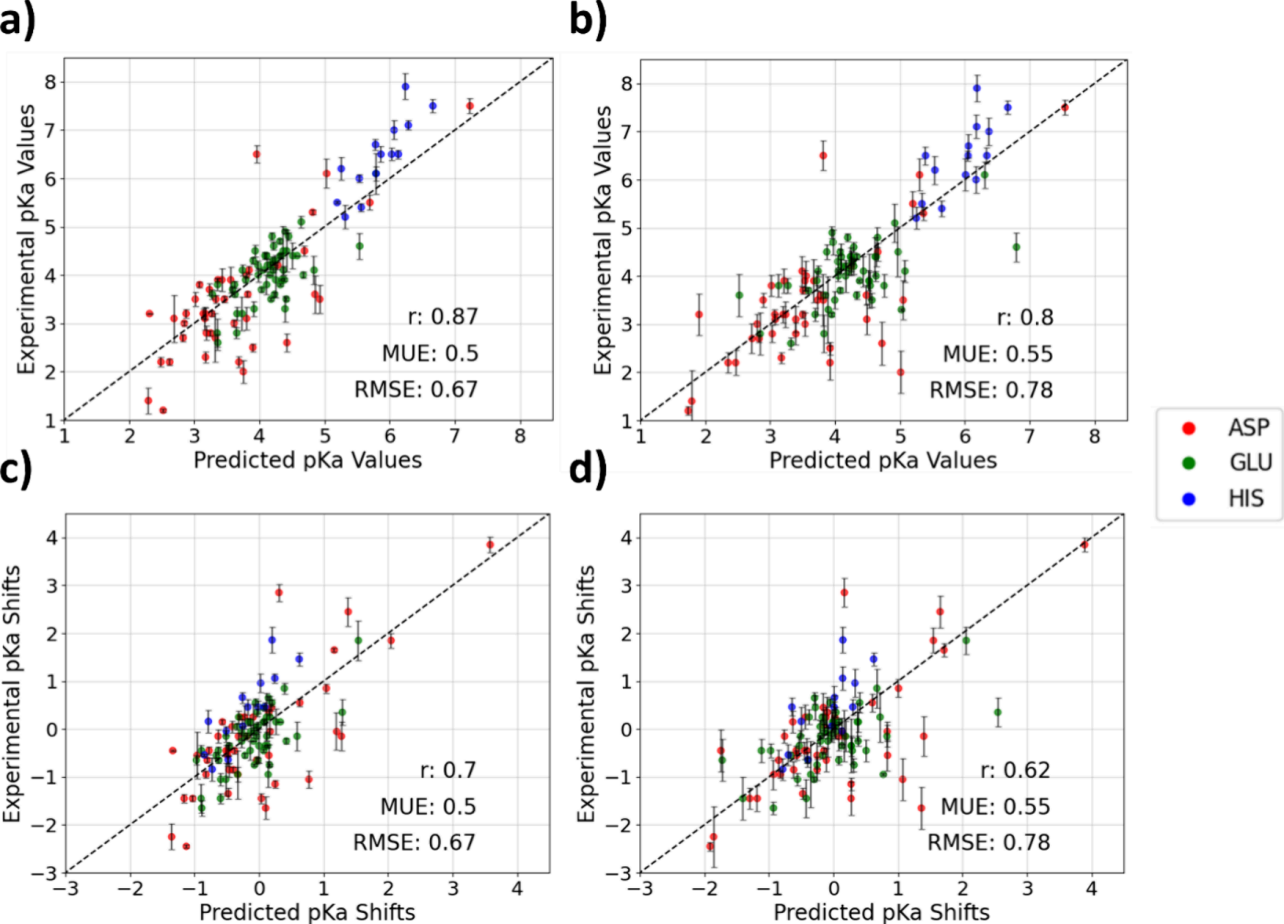
p*K*_a_ prediction results from pH-AFED combined with d-AFED for various proteins from (a) 10 ns of pH-AFED simulations at each pH and (b) 2 ns of pH-AFED simulations at each pH. Mean unsigned error (MUE), root mean squared error (RMSE), and Pearson correlation coefficient (*r*) are reported for each set. (c) Predicted vs experimental p*K*_a_ shifts (p*K*_a_–model amino acid p*K*_a_) using 10 ns of pH-AFED combined with d-AFED. (d) Predicted vs experimental p*K*_a_ shifts using 2 ns of pH-AFED combined with d-AFED. High p*K*_a_ shift of 4 represents that of Asp-26 of Thioredoxin, as highlighted in Figure 3.

Through combining d-AFED simulations with the pH-AFED scheme, we observed notably improved prediction metrics compared to the stand-alone pH-AFED with a MUE, RMSE, and *r* of 0.5, 0.67, and 0.86, respectively, in addition to overall smaller error bars between replicate simulations (Figure 4a). We also observed that pH-AFED, when combined with d-AFED, is able to provide predictive results from only very short simulation times of 2 ns (Figure 4b). Furthermore, we observed notable improvement in the prediction outlier of Glu-79 in Xylanase with a reduction in error from 1.88 p*K*_a_ units to 0.94 p*K*_a_ units. On the other hand, for the outliers Asp-21 of SNase and Asp-14 of RNaseA, we observed only marginal improvements when incorporating d-AFED with a reduction in error from 2.9 to 2.5 and 2.08 to 1.75, respectively. We also note that for all three residues, the incorporation of d-AFED showed significant improvements in the torsion sampling. These errors may, therefore, be attributed to inaccuracy from the force field in describing the complex electrostatics that may be present in buried or coupled residues. For instance, the residues of Asp-19/Asp-21 and Glu-75/His-121of SNase, or His-127/Glu-119 in RNaseH are coupled such that sufficiently describing the dynamics involving coupled electrostatics proved to be challenging using this approach, where >1 p*K*_a_ unit errors were observed for Asp-21, Glu-75, and His-127. In addition, other slower degrees of motion that are not sufficiently captured through enhancing of just the torsion angles of the titratable residues may also play an important role in accurately describing the ionization dynamics. Other CVs could be used in conjunction with the hybrid pH-AFED/d-AFED scheme. Further improvement of the accuracy of this approach may be achieved by combining pH-AFED with polarizable force fields^47^ or neural network potentials^48^ to describe intermolecular energetics more precisely or by employing more advanced machine learning approaches for rationally selecting CVs.^49,50^

### Comparisons with Other Methodologies

When compared to other state of the art all-atom CpHMD implementations in AMBER and CHARMM, we observed comparable predictive accuracy using pH-AFED and pH-AFED/dAFED, with shorter simulation times and fewer pH replicas (Table S9).^26,31^ In addition, in comparison to the widely utilized empirical approach, PROPKA3,^10^ our study demonstrates that the pH-AFED/d-AFED approach offers more robust and precise predictive power. While PROPKA3 achieves good predictive accuracy when applied to the RCSB deposited experimental structures of the proteins in our data set (MUE: 0.57, RMSE: 0.76, R: 0.84) (Table S5 ESI), its performance notably worsens when applied to the MD equilibrated structures from the same set (MUE: 0.95, RMSE: 1.27, R: 0.66) (Table S6 ESI). This discrepancy suggests the limitations of conventional static approaches in accurately predicting ionization behavior in disordered crystal structures or those with inadequate resolution. Consequently, our findings suggest the advantages of utilizing the hybrid pH-AFED/d-AFED approach, particularly when dealing with structural proposals obtained through Cryo-EM^51,52^ or AlphaFold,^53^ where atomic coordinates may not be perfectly resolved.

### Application to Therapeutic Antibodies

**Figure 5:**
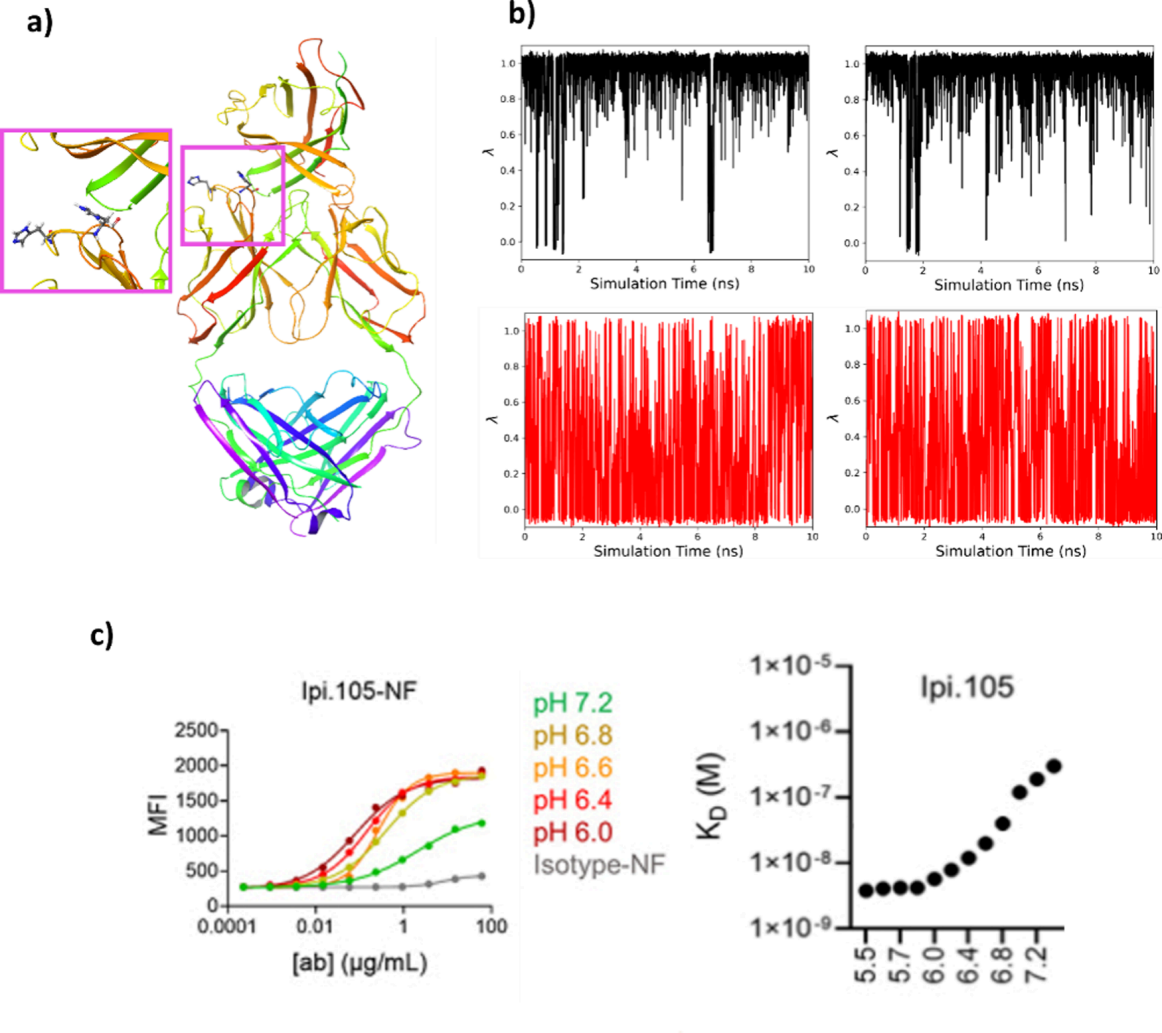
(a) Depiction of His-31 and His-55 on the IPI-105 and CTLA-4 complex. (b) λ trajectory of standard constant pH MD in black and pH-AFED/d-AFED in red at pH 7. (c) Adapted from ref (7) (left) pH-dependent cell binding for IPI-105 (right) pH-dependent SPR binding affinities to CTLA-4 of IPI-105.

Lastly, we utilized the hybrid pH-AFED/d-AFED approach on Ipilimumab (IPI), a marketed monoclonal antibody used for immunotherapy in various cancers through targeting CTLA-4 within tumor cells. Despite its efficacy, IPI exhibits dose-limiting toxicity, limiting its efficacy in certain patients. Here, we utilized our approach to study the engineered variant of Ipilimumab (IPI-105),^7^ reported by Lee et al., to better understand its toxicity and efficacy profile. IPI-105 is designed to bind to CTLA-4 preferentially at low pH, thereby increasing its activity within acidic tumors, while exhibiting weak binding at neutral pH in microenvironments of healthy tissue. From the analysis of the deposited crystal structure of the IPI-105, the heavy chain complementarity-determining regions (CDR) of IPI-105 exhibit histidine substitutions (His-55 and His-31) (Figure 5a) that interact with the CTLA-4 epitope. It is worth noting that these two histidine residues on IPI-105 are the mutated residues from IPI, the original monoclonal antibody. Given the intrinsic p*K*_a_ of histidine, which is close to physiologically relevant pH, the substituents present in IPI-105 around these histidine residues can modulate its pH-dependent binding activity to CTLA-4. This modulation occurs through alterations in the dynamic chemical environment experienced by each histidine, which can affect their ionization states. Consequently, the dynamic chemical environments exhibited by these histidine residues can significantly impact the pH dependent binding activity of IPI-105 to CTLA-4. Therefore, the use of constant pH MD may provide valuable insights into the ionization states of these residues.

When using the standard constant pH MD approach to study the IPI-105 system, we observed infrequent transitions within a 10 ns time frame (Figure 5b, black). As a result, the overall p*K*_a_ prediction for His-31 and His-55 were 8.05 and 8.23, respectively, indicating limited pH dependence at physiologically relevant pH levels. To overcome this sampling limitation, we utilized our hybrid enhanced sampling approach to study the dynamic ionization of the IPI-105 and CTLA-4 complex. This complex consists of over 50 titratable residues and over 100 torsional collective variables, which we can effectively explore using pH-AFED and d-AFED to enhance the sampling efficiency. Using this approach, which achieves rapid sampling of ionization states of the two residues (Figure 5b, red), the p*K*_a_ of His-31 and His-55 were determined to be 6.59 and 6.49, respectively. While there are no experimental p*K*_a_ values for this system, the predicted p*K*_a_ values align well with the observed pH dependent activity data as shown in Figure 5c,d. The results from the pH-AFED and d-AFED simulations confirm the structural hypothesis proposed by Lee et al.,^7^ highlighting the significance of this approach in providing valuable insights for optimizing antibody design and enhancing their therapeutic efficacy.

## Conclusions and Future Applicability

In summary, we show that the pH-AFED approach, which involves incorporation of a large *m*_λ_ for titratable residues while using independent thermostats to maintain titratable residues at a high *T*_λ_, achieves both improved sampling and predictive accuracy compared to standard CpHMD approaches. Here, we validated our approach on a range of proteins, where we observed reasonable agreement to experimental values with a MUE and RMSE of 0.61 and 0.81, respectively. In addition, pH-AFED allows enhanced sampling of protonation states without requiring multiple parallel replicas nor the exchange of configurations between different replicas. As such, this allows other enhanced sampling techniques to be easily implemented concurrently. We show that combining both enhanced sampling from d-AFED to drive selected torsion angles, with pH-AFED to enhance the sampling of the titration coordinate, we observed improved prediction accuracies with a MUE and RMSE of 0.50 and 0.68, respectively. Additionally, we showcase the practical application of this hybrid enhanced sampling approach on the engineered antibody, IPI-105, demonstrating how the approach enables simulation-based antibody design and in silico characterization with higher throughput, eliminating the need for very long simulation times.

Overall, the straightforward integration of the pH-AFED scheme into existing CpHMD implementations presents a promising direction for both macromolecule p*K*_a_ predictions and the investigation of proton coupled dynamics. In particular, this approach holds value in studying phenomena or structures where the ionized groups are strongly interacting or buried, such as transmembrane peptides or disproportionation in ionic crystal structures, through more efficient sampling. However, we note that accurately capturing the complex electrostatic interactions of strongly interacting or buried systems will also require more advanced potential energy models. Furthermore, pH-AFED simulations can be easily integrated with pH-replica exchange approaches in future studies, enhancing the overlap and acceptance ratios between adjacent replicas or also reformulated to efficiently study pH-coupled redox processes.^54^ Lastly, the versatility of pH-AFED allows for easy combination with enhanced sampling approaches, enabling the study of longer time scale proton-coupled processes such as membrane permeation or molecular assembly of macromolecular aggregates or ionizable lipid nanoparticles.

The version of pH-AFED and input files used to generate the results in this manuscript are available on Zenodo: https://zenodo.org/records/13924511.

## Computational Details

### MD Simulations and System Preparation

The simulated proteins in this study were placed in a 10 × 10 × 10 nm^3^ box of CHARMM TIP 3p water molecules. Na^+^ and Cl^–^ ions were added to all systems at a concentration of 0.15 M to neutralize the proteins. All MD simulations were performed using the GPU compatible GROMACS CpHMD implementation described by Aho et al.^28^ Coulombic interactions were described using the smooth PME method with a real space cutoff of 1.2 nm and a grid spacing of 0.14 nm, while the Lennard-Jones interactions are smoothly switched to zero from 1.0 to 1.2 nm. A leapfrog integrator was used to integrate both the molecular and lambda coordinates with an integration step of 2 fs. The LINCs algorithm was used to constraint the hydrogen bonding lengths of the proteins and the SETTLE algorithm was used to constrain the degrees of freedom of the water molecules.^55,56^ Titratable buffers, as described by Donnini et al. were used to maintain charge neutrality where 10 titratable buffers are added for each titratable residue.^39^ MD simulations were performed using the CHARMM36 force field with torsion modifications, as described by Buslaev et al. to enhance conformational sampling.^57,58^ Constant pressure was maintained at 1 bar using the Parrinello–Rahman Barostat with relaxation times of 2.0 ps. All systems were equilibrated for 2 ns before the production CpHMD or pH-AFED simulations. The following reference p*K*_a_ values were used in the CPHMD simulations: Glu: 4.25, ASP: 3.65, His Tautomer 1: 6.53, His Tautomer 2: 6.92. However, we note here, there may be deviations from experimental values due to offsets in the absolute value in NMR experiments.^59^ Hydrogen bond assessments were conducted using the GROMACS gmx hbond module, which defines hydrogen bonds using the criteria that donor and acceptor atoms are within 3.5 Å, with a hydrogen bond donor–acceptor angle less than 30°.^60^

### Standard CpHMD Simulations

The standard CpHMD simulations were performed using the approaches described by Aho et al. where Asp and Glu residues are described using a single site representation, and His residues are described using a multisite representation. In this, approach a global velocity-rescale thermostat was used to control both the temperature of the molecular coordinates and lambda coordinates at 300 K with a time constant of 0.5 ps^–1^ and a *m*_λ_ of 5 au.^61^

### pH AFED Simulations

Like the standard CpHMD approach, single site representations are used to describe Asp and Glu and a multisite representation is used to describe His. In this approach each individual titration coordinate is assigned a Nosé–Hoover Chain thermostat (chain length = 4, time constant = 0.5 ps^–1^, Suzuki-Yoshida order = 4, multiple time step = 5).^63,64^ The λ coordinates are propagated using the velocity-verlet algorithm with a “side” thermostatting scheme.^38^

To compute the p*K*_a_ values using pH-AFED, multiple simulations are conducted at different pH conditions, and the probability of deprotonation is determined through eq 8. For single site titratable residues of the acids Glu and Asp, for each frame of the simulation, we consider a site deprotonated if λ > 0.8 and protonated if λ < 0.2. In eq 8, *n*_prot_pHAFED_ and *n*_deprot_pHAFED_ denote the number of protonated and deprotonated frames observed in the pH-AFED trajectory at temperature *T*_λ_, while *n*_prot_ and *n*_deprot_ denote the computed protonation and deprotonation ratios expected to be observed at temperature *T*, or typically, 300 K.
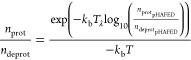
8
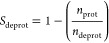


For His, which utilizes a multisite representation to describe the ionization state (eq 9), where *N*_p_ denotes the protonated state of His, and *N*_c_ and *N*_δ_ denote the different neutral tautomeric states of His. Here, for the His base, we consider a site protonated if λ > 0.8 and deprotonated if λ < 0.2. The deprotonation fraction can then be computed using eq 9. For His.
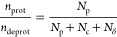
9

The deprotonation fractions are then fit using the generalized Henderson–Hasselbalch equation in eq 10:
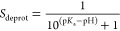
10

The coupled residues of Asp-19 and Asp-21 were also fit using a coupled titration model in eq 11, as described by Harris et al. where <*P*> describes the average number of protons between two coupled residues. The results, however, were determined to be almost identical to those from using eq 10.
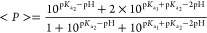
11

### d-AFED Enhanced Sampling

d-AFED enhanced sampling was performed using the PLUMED plugin for GROMACS.^62^ In this implementation of d-AFED, the temperature is controlled through a Langevin thermostat with *k* = 1000 kJ/mol, *T*_s_ = 1000 K, mass = 20 au/(nm/rad)^2^, γ (friction) = 5.0, τ = 0.1 p.s. The following were used as collective variables for all titratable residues that exhibit a SASA of less than 80 Å^2^: The following torsion angles were used as collective variables for ASP Residues: N–C_γ_–C_β_–C_α_, O_α_–C_δ_–C_α_–C_β_, and H–O_δ2_–C_δ_–O_δ1_ torsion angles; for GLU Residues: N–C_γ_–C_β_–C_α_, C_δ_–C_α_–C_β_–C_γ_, O_δ1_–C_δ_–C_β_–C_α_, and H–O_δ2_–C_δ_–O_δ1_ torsion angles; for HIS Residues: N–C_α_–C_β_–C_γ_, and H–N–C_α_–C_β_.

## Supporting Information Available

Equations and parameters for the barrier and pH potentials used in simulations, outline and equations of Adiabatic Free Energy Dynamics (AFED), temperature control using massive thermostats in the pH-AFED scheme, predictions of HEWL p*K*_a_ using various pH AFED *T*_λ_ and *m*_λ_ parameters, convergence plots showing deprotonation fractions of HEWL for pH-AFED and GROMACS CPHMD, table of p*K*_a_ values for benchmark proteins using pH-AFED, table of p*K*_a_ values for benchmark proteins using standard GROMACS CpHMD, table of p*K*_a_ values for benchmark proteins from combining pH-AFED with d-AFED, description of quantification of mixed states using pH-AFED, table of mixed lambda states with different pH-AFED parameters for each system, table of p*K*_a_ values for benchmark proteins using pH-AFED with a larger barrier potential, p*K*_a_ values for benchmark proteins obtained from PROPKA3, table of p*K*_a_ values for benchmark proteins of MD equilibrated structures using PROPKA3, torsional analysis for pH-AFED, table comparing pH-AFED performance with reported results of other all-atom CpHMD implementations, and figure depicting torsion angles enhanced using d-AFED (PDF)
