# Supplementary material for: Enhanced and Efficient Predictions of Dynamic Ionization through Constant-pH Adiabatic Free Energy Dynamics
Source: J Chem Theory Comput. 2024 Nov 8;20(22):10010–21. doi: 10.1021/acs.jctc.4c00704 (PMC11603612; doi:10.1021/acs.jctc.4c00704)
Supplement: Supplementary file 1 — ct4c00704_si_001.pdf [file ct4c00704_si_001.pdf]

Enhanced and Efficient Predictions of Dynamic Ionization through Constant-pH Adiabatic Free Energy Dynamics

Richard S. Hong [a, b], Busayo D. Alagbe [a] , Alessandra Mattei [a], Ahmad Y. Sheikh [a], Mark E. Tuckerman [b, c, d,e*]

[a] AbbVie Inc., Molecular Profiling and Drug Delivery, Research & Development, 1 N Waukegan Road, North Chicago, IL 60064, United States

[b] Department of Chemistry, New York University, New York City, New York 10003, United States

[c] Courant Institute of Mathematical Sciences, New York University, New York, NY 10012, USA

[d] NYU-ECNU Center for Computational Chemistry at NYU Shanghai, 3663 Zhongshan Road North, Shanghai 200062, China

e Simons Center for Computational Physical Chemistry at New York University, New York, New York 10003, United States

Barrier and pH Potentials Used for Constant pH MD

Below are pH potential and Barrier potential equations used for each individual λ coordinate:

pH Potential:

$$V_{Base}^{pH}(\lambda) = RT\, log_{10}\,(pKa - pH)\left(\frac{1}{1 + \exp\left(-2k_i(\lambda_i - 1 + x_o)\right)}\right) \qquad (1)$$

$$V_{Acid}^{pH}(\lambda) = RT\, log_{10}\,(pKa - pH)\left(\frac{1}{1 + \exp\left(-2k_i(\lambda_i - x_o)\right)}\right) \qquad (2)$$

Barrier Potential:

To ensure sampling of λ at physically relevant ionization states (where λ =0 or λ =1), a barrier potential $V^{barrier}(\lambda)$, described by Eq. 3, is utilized. The terms in Eq. 3 are solved iteratively, following the approach described by Aho et. al., given a desired barrier height.[1]

$$V^{barrier}(\lambda) = -k\left[\exp\left(-\frac{(\lambda - 1 - \mathrm{b})^2}{2a^2}\right) + \exp\left(-\frac{(\lambda + \mathrm{b})^2}{2a^2}\right)\right] + d\left[\exp\left(-\frac{(\lambda - 0.5)^2}{2s^2}\right)\right]$$
$$+\ 0.5w((1 - \mathrm{erf}[r(\lambda + \mathrm{m})]) + (1 + \mathrm{erf}[\mathrm{r}(\lambda - 1 - \mathrm{m}])) \qquad (3)$$

Bond Potential:

Lastly, $V^{bond}(\lambda)$, a polynomial function representing a correction term for the quantum mechanical contributions of protonation and deprotonation is incorporated in the overall potential energy. The forces origination from this term, $\frac{d\mathrm{V}^{bond}}{d\lambda}$, are expressed as a polynomial function (Eq. 4) with the coefficients, a_k, determined by evaluating the deprotonation free energy in water. To achieve this, the trajectory averages, $< \frac{d\mathrm{V}}{d\lambda} >_\lambda$ are initially computed for each simulation, allowing for the subsequent acquisition of polynomial coefficients through a polynomial fit of $\frac{d\mathrm{V}^{bond}}{d\lambda}$ from these trajectory averages.

$$\frac{dV^{bond}}{d\lambda} = \sum_{k=0}^{n} a_k x^k \qquad (4)$$

The following $\frac{dV^{bond}}{d\lambda}$ coefficients were utilized for the residues included in this study, similar to those used in Ref 1:

ASP: 69.73, -457.77, 530.42, -215.67, -551.97, 44.21

GLU: 39.85, -375.98, 462.20, -185.00, -560.54, 26.79

HIS (Protonated): -3015.4, -13444.1, -34779, -44335.5, -31383.1, -13684.4, -4905.57, 106.148, -484.107, 11865.5, 48985.4, 104163, 103612, 59628.7, 25794.9, 3490.9, 2180.34, -18552.6, -70083.6, -117174, -91492.5, -47959.2, -12542.4, -3968.74, 14751.8, 49997.8, 63417.3, 42048.3, 16441.1, 3929.98, -6193.44, -18113.5, -17591.7, -9832.74, -2246.33, 934.809, 2225.21, 1887.54, 594.263, 402.485, 604.077, 158.757, -463.471, -193.458, 82.6296

HIS (Tautomer 1): -1695.49, -8107.68, -22614.3, -40090.6, -39319.4, -21686.8, -5816.32, -1663.43, -1231.35, 7080.8, 30409.5, 75205.7, 105063, 78113.9, 30391.7, 8898.15, 5178.6, -12000.8, -45996.4, -95996.2, -101472, -54848.1, -18185.2, -8896.93, 10595.2, 35922, 59318.7 45645, 18480.3, 8030.77, -5015.37, -14771.9, -17941.8, -9642.21, -3996.01, 894.376, 2282.28, 1905.97, 857.662, 264.085, 372.239, 128.992, -135.244, -299.418, -28.5093

HIS (Tautomer 2): -111.044, -768.566, -9092.12, -17291.1, -2883.78, 15464.2, 13669.9, 5673.64, 433.027, 873.492, 5534.28, 29537.7, 27498.6, -17935.7, -31834.8, -17251.3, -1555.29, -2245.47, -11551.3, -31061.6, -4912.98, 24459.9, 19180.8, 2258.94, 2451.91, 9281.43, 10136.1, -7134.35, -9948.52, -1755.79, -986.264, -1997.06, 1570.57, 3175.52, 958.098, -64.3349, -650.924, -1045.66, -389.613, -6.33165, 63.2438, 4.25894, 286.277, 228.063, -171.139

Adiabatic Free Energy Dynamics

The groundwork for the pH AFED approach is based on the principles of the original Adiabatic Free Energy Dynamics methodology.[2-3] Here, like the approach described previously, a barrier potential in the λ free energy space is introduced to promote sampling at physically relevant λ states (close to 0 or 1). To ensure that the introduction of this barrier does not hinder sampling of the end point λ states, a high temperature T_λ is assigned to the dynamic λ variable. In addition, a large mass term m_λ is introduced to ensure that the dynamics of the λ variable is adiabatically decoupled from the physical system. From this, the free energy profiles of the λ states can be recovered through the probability distribution of λ sampled throughout the simulation with the form:

$$F(\lambda) = -k_b T_\lambda \ln P(\boldsymbol{\lambda})$$

The Hamiltonian of the adiabatic free energy dynamics approach, including that of pH-AFED can be expressed as the following:

$$H(\boldsymbol{r}, \boldsymbol{\lambda}) = \frac{\boldsymbol{p}_{\boldsymbol{r}}^2}{2\boldsymbol{M}_{\boldsymbol{r}}} + \frac{\boldsymbol{p}_{\boldsymbol{\lambda}}^{\boldsymbol{2}}}{2\boldsymbol{M}_{\boldsymbol{\lambda}}} + V(\boldsymbol{r}, \boldsymbol{\lambda})$$

The Liouville operator for the thermostatted dynamics can then be expressed as the following:

$$iL = \frac{1}{\boldsymbol{M}_{\boldsymbol{r}}}\boldsymbol{p}_{\boldsymbol{r}}\frac{d}{d\boldsymbol{r}} + \frac{\boldsymbol{p}_{\boldsymbol{\lambda}}}{\boldsymbol{M}_{\boldsymbol{\lambda}}}\frac{d}{d\boldsymbol{\lambda}} + \boldsymbol{F}_{\boldsymbol{r}}\frac{d}{d\boldsymbol{r}} + \boldsymbol{F}_{\boldsymbol{\lambda}}\frac{d}{d\boldsymbol{\lambda}} + iL_{therm}^{(\boldsymbol{r})}(T) + iL_{therm}^{(\boldsymbol{\lambda})}(T_s)$$

where $\boldsymbol{r} = r_i \ldots . r_N$, $\boldsymbol{p}_{\boldsymbol{r}} = p_i \ldots . p_N$, $\boldsymbol{p}_{\boldsymbol{\lambda}} = p_{\lambda i} \ldots . p_{\lambda n}$, $\boldsymbol{F}_{\boldsymbol{r}} = F_i \ldots . F_N$, $\boldsymbol{F}_{\boldsymbol{\lambda}} = F_{\lambda i} \ldots . F_{\lambda n}$, $\boldsymbol{M}_{\boldsymbol{r}} = m_i \ldots m_N$, $\boldsymbol{M}_{\boldsymbol{\lambda}} = m_{\lambda 1} \ldots m_{\lambda n}$, for N atoms in the physical system and n titratable residues, $iL_{therm}^{(r)}(T)$ corresponds to the thermostat for the physical system, $iL_{therm}^{(\lambda)}(T_\lambda)$ corresponds to the thermostat for the dynamical λ variable. Here, we see that there are two separate thermostats for controlling the temperatures of the physical system and the dynamical λ variable.

The time evolution of the full phase space, starting from an initial condition $\Gamma(0)$ can be expressed as:

$$\Gamma(t) = e^{iLt}\,\Gamma(0)$$

where e^{iLt} is the classical propagator. Here, we consider the evolution of the system over a time interval Δt characteristic of the λ dynamics. To factorize the propagator in a way consistent to analyze the dynamics over Δt consistent with adiabatic decoupling, we define the following:

$$iL_{\boldsymbol{r}}^{(ref)} = \frac{1}{\boldsymbol{M}_{\boldsymbol{r}}}\boldsymbol{p}_{\boldsymbol{r}}\frac{d}{d\boldsymbol{r}} + iL_{therm}^{(\boldsymbol{r})}(T)$$

$$iL_{\boldsymbol{\lambda}}^{(ref)} = \frac{\boldsymbol{p}_{\boldsymbol{\lambda}}}{\boldsymbol{M}_{\boldsymbol{\lambda}}}\frac{d}{d\boldsymbol{\lambda}} + iL_{therm}^{(\lambda)}(T_\lambda)$$

$$iL_{\boldsymbol{r}} = iL_{\boldsymbol{r}}^{(ref)} + \boldsymbol{F}_{\boldsymbol{\lambda}} \frac{d}{d\boldsymbol{p}_{\boldsymbol{\lambda}}} + \boldsymbol{F}_r \frac{d}{d\boldsymbol{p}_{\boldsymbol{r}}}$$

With the total Liouville operator given by

$$iL = iL_r + iL_{\boldsymbol{\lambda}}^{(ref)} + iL_{\boldsymbol{r}}^{(ref)}$$

Now, the Trotter theorem can be used to construct a symplectic, reversible factorization for the classical propagator:

$$\exp(iL\Delta t) = \exp\,(\frac{iL_{\boldsymbol{r}}\Delta t}{2})\exp\,(iL_{\boldsymbol{\lambda}})\exp\,(\frac{iL_{\boldsymbol{r}}\Delta t}{2})$$

Here, the operator of the physical system $\exp\,(\frac{iL_r\Delta t}{2})$ has terms that vary on a time scale much faster than Δt (the time scale at which λ evolves). Under adiabatic conditions, the time interval Δt is much longer than the time scale at which **r** evolves. Hence, it must be further decomposed to the following:

$$\exp\left(\frac{iL_{\boldsymbol{r}}\Delta t}{2}\right) = \lim_{n\to\infty}\left[\exp\left(\frac{\Delta t}{4n}\boldsymbol{F}_{\boldsymbol{\lambda}}\frac{d}{d\boldsymbol{p}_{\boldsymbol{\lambda}}}\right)\exp\left(\frac{\Delta t}{4n}\boldsymbol{F}_r\frac{d}{d\boldsymbol{p}_{\boldsymbol{r}}}\right)\exp\left(\frac{iL_{\boldsymbol{r}}^{(ref)}\,\Delta t}{2n}\right)\exp\left(\frac{\Delta t}{4n}\boldsymbol{F}_{\boldsymbol{r}}\frac{d}{d\boldsymbol{p}_{\boldsymbol{r}}}\right)\exp\left(\frac{\Delta t}{4n}\boldsymbol{F}_{\boldsymbol{\lambda}}\frac{d}{d\boldsymbol{p}_{\boldsymbol{\lambda}}}\right)\right]^n$$

Due to the large mass of λ, where $\boldsymbol{M}_{\boldsymbol{\lambda}} \gg \boldsymbol{M}_{\boldsymbol{r}}$**,** the motion of r will rapidly follow the motion of r and sample its available phase space over the time interval $\frac{\Delta t}{2}$, where λ remains fixed. Here, the force for the λ variables, is related to a time average of F_λ over the adiabatic r trajectory:

$$\overline{F_\lambda} = \frac{2}{\Delta t}\int_{\tau}^{\tau+\frac{\Delta t}{2}} dt\; F_\lambda(r(t; x(\tau)), \lambda)$$

Here, x(τ) is the full phase space vector at time τ. Here, F_λ is determined at a fixed λ but time varying r. If enough of the phase space of r is sampled over Δt, the following can be approximated where the time average can be replaced by a phase space average:

$$\frac{2}{\Delta t}\int_{\tau}^{\tau+\frac{\Delta t}{2}} dt\; F_\lambda(\boldsymbol{r}(t; x(\tau)), \boldsymbol{\lambda}) = \frac{d}{d\boldsymbol{\lambda}}\frac{1}{\beta}\ln\, Z_r(\boldsymbol{\lambda}, \beta)$$

Where:

$$Z_r(\boldsymbol{\lambda}, \beta) = \int d\boldsymbol{r}\, e^{-\beta V(r,\boldsymbol{\lambda})}$$

defines the configurational partition function at fixed values of the λ. An effective Hamiltonian for the titratable coordinates can then be defined as

$$H(\boldsymbol{\lambda}, \boldsymbol{p}_{\boldsymbol{\lambda}}) = \frac{\boldsymbol{p}_{\boldsymbol{r}}^{\mathbf{2}}}{2\boldsymbol{M}_{\boldsymbol{r}}} - \frac{1}{\beta} \ln Z_r(\boldsymbol{\lambda}, \beta)$$

Assuming the dynamics are adiabatically decoupled, thermostats applied to this Hamiltonian will give the canonical distribution at the temperature T_λ and the probability distributions:

$$P_{adb}(\boldsymbol{\lambda}) = C\left[\int d\boldsymbol{p}_{\boldsymbol{r}} \exp\,(-\beta_\lambda\, \frac{\boldsymbol{p}_{\boldsymbol{r}}^{\mathbf{2}}}{2\boldsymbol{M}_{\boldsymbol{r}}})\right] \exp\left\{-\beta_{\boldsymbol{\lambda}}(-\frac{1}{\beta} \ln Z_r(\,\boldsymbol{\lambda}, \beta)\right\}$$

$$P_{adb}(\boldsymbol{\lambda}) = C\left[\int d\boldsymbol{p}_{\boldsymbol{r}} \exp\left(-\beta_\lambda\, \frac{\boldsymbol{p}_{\boldsymbol{r}}^{\mathbf{2}}}{2\boldsymbol{M}_{\boldsymbol{r}}}\right)\right] Z_r(\boldsymbol{\lambda}, \beta)^{\frac{\beta_{\boldsymbol{\lambda}}}{\beta}}$$

Since the free energy of $\boldsymbol{\lambda}$ is defined as

$$A(\boldsymbol{\lambda}) = \frac{1}{\beta} \ln Z_\lambda\, (\boldsymbol{\lambda}, \beta)$$

Using this probability distribution, $P_{adb}(\boldsymbol{\lambda})$, the free surface energy of $\boldsymbol{\lambda}$ can be determined with the following:

$$A(\boldsymbol{\lambda}) = -\frac{1}{\beta_\lambda} \ln P_{adb}(\boldsymbol{\lambda}) = -k_b T_\lambda \ln P_{adb}(\boldsymbol{\lambda})$$

where the free energy surface can be determined directly from the probability distribution function, $P_{adb}(\boldsymbol{\lambda})$, generated from an AFED MD trajectory.

Maintaining Temperature Control – Charge Constraints with Massive Thermostatting

Lambda Integration Scheme:

1. Apply Nosé-Hoover chain thermostat using half a timestep.
2. Lambda Velocity Verlet: Update lambda velocity half timestep, then update lambda positions full timestep.
3. Recompute forces on lambda variable.
4. Propagate lambda velocities a second half step.
5. Apply Nosé-Hoover chain thermostat using half a time step.
6. Apply "SHAKE" like charge constraints.

Coupling residues to titratable buffers:

Considering only charge constraints where (sum of total charges is equal to 0):

$$(\mathbf{1})\quad \sigma(\lambda) = \sum_{i=1}^{N_{sites}} \alpha_i \lambda_i = 0$$

Here, λ_i is the charge coordinate, and α_i represents the total charge of the residue where:

$$(\mathbf{2})\quad \alpha_i = \sum_{j}^{N_{atoms}^{i}} q_{j,i}^{deprot} - q_{j,i}^{prot}$$

Assume for a simple system for 1 titratable ASP residue constrained to 10 collective buffer particles:

$$(\mathbf{3})\quad \sigma(\lambda) = \alpha_{ASP}\lambda_{ASP} + \alpha_{Buf}\lambda_{Buf} = 0$$

Here, for the ASP residue (charge difference between the protonated vs the deprotonated species is 1):

$$(\mathbf{4})\quad \alpha_{ASP} = \sum_{j}^{N_{atoms}^{ASP}} q_{j,ASP}^{deprot} - q_{j,ASP}^{prot} = 1$$

For the BUF residue (charge difference between the protonated vs the deprotonated species is 10, for 10 collective buffer particles):

$$(\mathbf{5}) \quad \alpha_{Buf} = \sum_{j}^{N_{atoms}^{BUF}} q_{j,Buf}^{deprot} - q_{j,Buf}^{prot} = 10$$

The unconstrained λ^{u} coordinates after each time step are corrected to the constrained λ^{u} coordinates through constraint forces, $F_i = -\zeta \frac{d\sigma}{d\lambda_i}$, F_{ASP} and F_{BUF} :

$$(\mathbf{6}) \quad \frac{d\sigma}{d\lambda_i} = \alpha_i$$

$$(\mathbf{7})\ \lambda_{ASP}^{c} = \lambda_{ASP}^{u} + \frac{F_{ASP}}{2m_{ASP}}\Delta t^2 = \lambda_{ASP}^{u} - \zeta\frac{\alpha_{ASP}}{2m_{ASP}}\Delta t^2$$

$$(\mathbf{8})\ \lambda_{BUF}^{c} = \lambda_{BUF}^{u} + \frac{F_{BUF}}{2m_{BUF}}\Delta t^2 = \lambda_{BUF}^{u} - \zeta\frac{\alpha_{BUF}}{2m_{BUF}}\Delta t^2$$

The velocities are then affected through Eq. 9 and Eq. 10

$$(\mathbf{9}) \quad \dot{\lambda}_{ASP}^{c} = \dot{\lambda}_{ASP}^{u} - \zeta\frac{\alpha_{ASP}}{m_{ASP}}\Delta t$$

$$(\mathbf{10}) \quad \dot{\lambda}_{BUF}^{c} = \dot{\lambda}_{BUF}^{u} - \zeta\frac{\alpha_{BUF}}{m_{BUF}}\Delta t$$

Since $\alpha_{BUF} = 10\ \alpha_{ASP}$, and $\dot{\lambda}^2 \propto T_{\lambda}$, the effect of massive thermostats coupled with charge - constraints on the velocities and temperature of the titratable site is considerably less that than on the velocities of the buffer. As such, this will have minimal impact on the free energies of the non-buffer, titratable residues.

Table S1. Predictions of HEWL using different T_λ and m_λ

Residue	pH-AFED (10 ns) T_λ=1000 m_λ = 1000	pH-AFED (10 ns) T_λ=500 m_λ = 500	Exp.
Glu-7	3.45	3.11	2.6
His-15	4.96	5.32	5.5
Asp-18	3.65	3.54	2.8
Glu-35	5.98	7.06	6.1
Asp-48	2.31	1.91	1.4
Asp-52	4.92	5.26	3.6
Asp-66	2.82	2.17	1.2
Asp-87	2.89	2.66	2.2
Asp-101	4.5	4.57	4.5
Asp-119	3.08	3.28	3.5
**MUE**	0.73	0.63	-
**RMSE**	0.87	0.77	-

Convergence Plots of Deprotonated Fractions for Each System using pH-AFED and Standard GROMACS CpHMD

HEWL pH-AFED

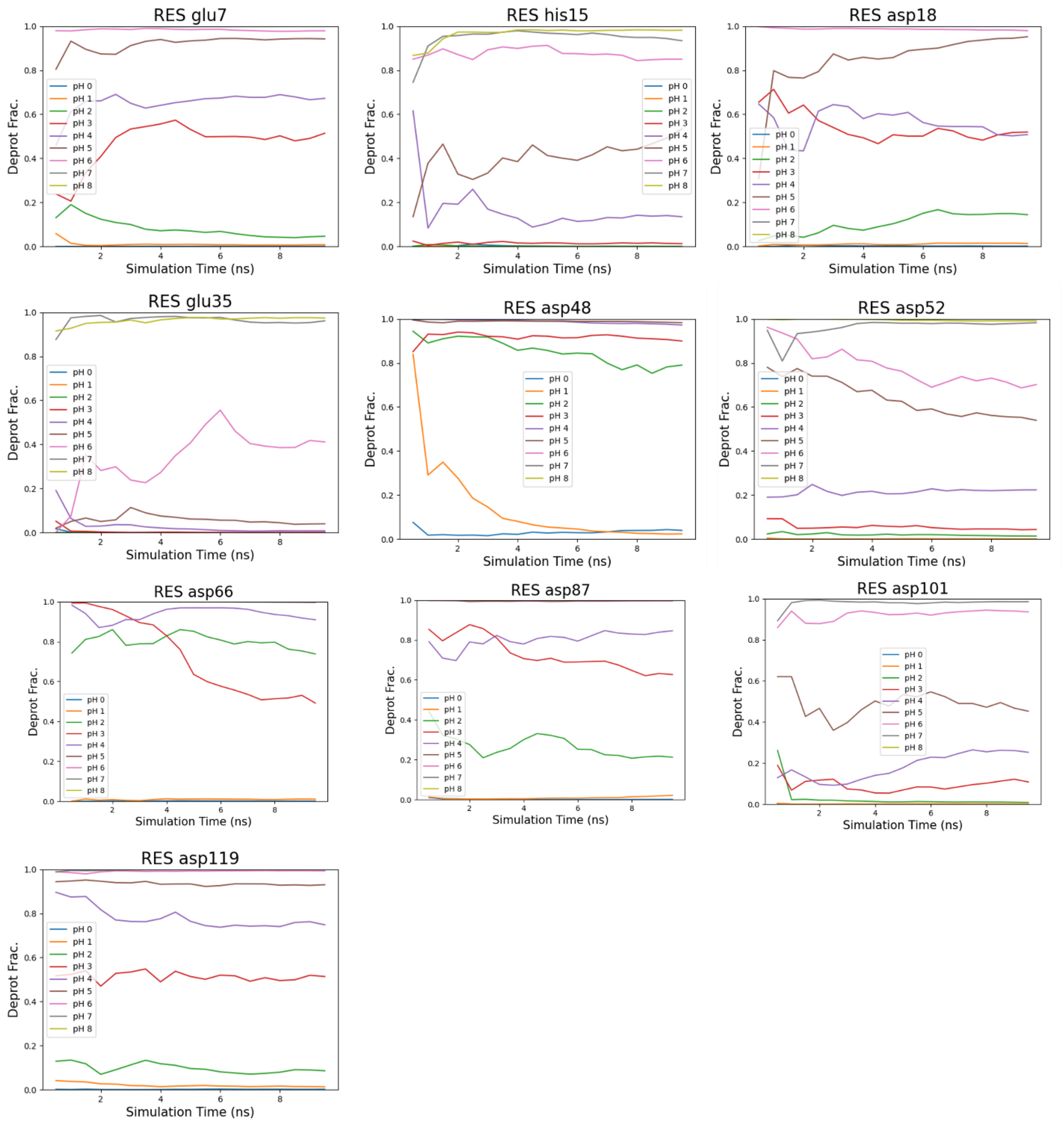

Figure S1. Convergence Plots of Deprotonated Fractions for Each System using pH-AFED at various pH

HEWL Standard GROMACS CPHMD

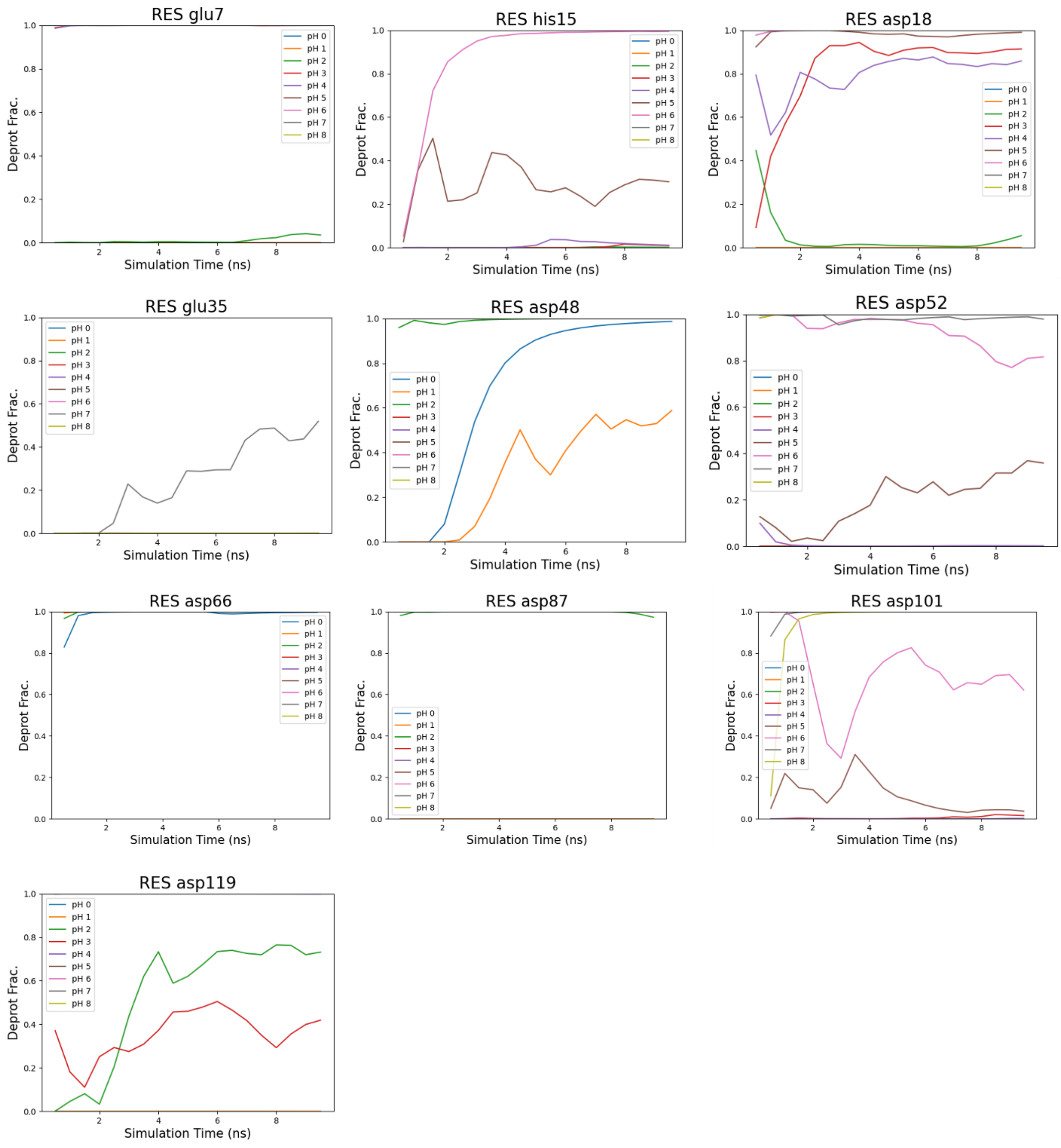

Figure S2. Convergence Plots of Deprotonated Fractions for Each System using Standard GROMACS CPHMD at various pH conditions. Empty convergence plots with a flat line may signify no lambda transitions have occurred during the trajectory, hence the absence of any sampling.

Table S2. Table of pK_a values for benchmark proteins of residues with available experimental data and predicted pK_a values from ***pH-AFED***

**HEWL**	**Exp**	**pH-AFED**		**SNase**	**Exp**	**pH-AFED**		**BBL**	**Exp**	**pH-AFED**
Glu7	2.6	3.27		His8	6.5	6.25		Asp129	3.9	3.34
His15	5.5	5.14		Glu10	2.8	3.76		Glu141	4.5	4.11
Asp18	2.8	3.13		Asp19	2.2	2.79		His142	6.5	5.83
Glu35	6.1	6.49		Asp21	6.5	3.61		Asp145	3.7	3.11
Asp48	1.4	2.07		Asp40	3.9	3.51		Glu161	3.7	4.11
Asp52	3.6	5.03		Glu43	4.3	4.07		Asp162	3.2	1.98
Asp66	1.2	1.96		Glu52	3.9	4.30		Glu164	4.5	4.02
Asp87	2.2	2.75		Glu57	3.5	4.46		His166	5.4	4.94
Asp101	4.5	4.90		Glu67	3.8	4.66				
Asp119	3.5	3.01		Glu73	3.3	3.87				
				Glu75	3.3	4.43		**Xylanase**		
**OMTKY**				Asp95	2.2	3.68		Asp5	3	4.02
Asp8	2.7	2.93		Glu101	3.8	4.13		Asp12	2.5	4.05
Glu11	4.1	4.00		His121	5.2	5.86		Glu79	4.6	6.48
Glu20	3.2	3.66		Glu122	3.9	3.84		Asp107	2.7	2.80
Asp27	2.3	3.80		Glu129	3.8	3.98		Asp120	3.2	3.16
Glu44	4.8	4.48		Glu135	3.8	3.24		Asp122	3.6	3.68
His53	7.5	6.54						His157	6.5	6.00
				**RNaseA**						
**Thioredoxin**				Glu2	2.8	3.51		**RNaseH**		
Glu6	4.8	4.53		Glu9	4	4.92		Glu6	4.5	4.23
Glu13	4.4	4.42		His12	6.2	5.19		Asp10	6.1	4.72
Asp16	4	3.70		Asp14	2	3.81		Glu32	3.6	2.52
Asp20	3.8	3.08		Asp38	3.5	3.27		Glu48	4.4	4.79
Asp26	7.5	7.78		His48	6	6.83		Glu57	3.2	2.71
Glu47	4.1	4.35		Glu49	4.7	3.81		Glu61	3.9	3.93
Glu56	5.1	4.58		Asp53	3.9	3.53		His62	7	6.06
Asp58	5.3	5.03		Asp83	3.5	5.29		Glu64	4.4	4.15
Asp60	2.8	3.67		Glu86	4.1	4.72		Asp70	2.6	4.68
Asp61	4.2	4.28		His105	6.7	5.70		Asp83	5.5	5.75
Asp64	3.2	3.15		Glu111	3.6	4.12		Asp94	3.2	3.25
Glu68	4.9	4.00		His119	6.1	5.39		Asp108	3.2	2.79
Glu70	4.6	4.12		Asp121	3.1	4.40		Glu119	4.1	4.46
Glu88	3.7	3.58						His124	7.1	6.13
Glu95	4.1	3.92						His127	7.9	5.59
Glu98	3.9	4.46						Glu129	3.6	4.46
Glu103	4.4	4.30						Glu131	4.3	4.50
								Asp134	4.1	3.74
								Glu135	4.3	4.14
								Glu147	4.2	4.26
								Glu154	4.4	4.22

**NTL**	**Exp**	**pH-AFED**		
Asp8	3	3.07		
Glu17	3.6	3.80		
Asp23	3.1	3.26		
Glu38	4	4.21		
Glu48	4.2	3.54		
Glu54	4.2	4.04		
**HP36**				
Asp44	3.1	2.72		
Glu45	4	3.96		
Asp46	3.5	3.68		
Glu72	4.4	4.66		
**Overall RMSE:**				
HEWL	0.68			
OMTKY	0.77			
Thioredoxin	0.44			
SNase	0.98			
RNaseA	1.01			
BBL	0.64			
Xylanase	1.02			
RNaseH	0.88			
NTL	0.49			
HP36	0.25			

Table S3. Table of pK_a values for benchmark proteins of residues with available experimental data and predicted pK_a values from 10 ns of ***Standard GROMACS CpHMD***

**HEWL**	**Exp**	**Pred**		**SNase**	**Exp**	**Pred**		**BBL**	**Exp**	**Pred**
Glu7	2.6	2.60		His8	6.5	6.81		Asp129	3.9	3.11
His15	5.5	4.95		Glu10	2.8	3.57		Glu141	4.5	4.31
Asp18	2.8	3.07		Asp19	2.2	1.81		His142	6.5	5.93
Glu35	6.1	8.56		Asp21	6.5	10.62		Asp145	3.7	2.87
Asp48	1.4	0.37		Asp40	3.9	3.55		Glu161	3.7	4.31
Asp52	3.6	5.73		Glu43	4.3	4.02		Asp162	3.2	1.82
Asp66	1.2	-0.08		Glu52	3.9	4.82		Glu164	4.5	3.98
Asp87	2.2	2.14		Glu57	3.5	4.94		His166	5.4	4.77
Asp101	4.5	5.37		Glu67	3.8	5.12				
Asp119	3.5	2.57		Glu73	3.3	3.25				
				Glu75	3.3	6.69		**Xylanase**		
**OMTKY**				Asp95	2.2	3.39		Asp5	3	4.49
Asp8	2.7	2.22		Glu101	3.8	4.09		Asp12	2.5	3.90
Glu11	4.1	3.99		His121	5.2	4.92		Glu79	4.6	10.52
Glu20	3.2	3.39		Glu122	3.9	5.84		Asp107	2.7	2.41
Asp27	2.3	4.18		Glu129	3.8	3.91		Asp120	3.2	2.95
Glu44	4.8	5.00		Glu135	3.8	2.67		Asp122	3.6	4.02
His53	7.5	6.84						His157	6.5	6.81
				**RNaseA**						
**Thioredoxin**				Glu2	2.8	2.76		**RNaseH**		
Glu6	4.8	4.53		Glu9	4	5.50		Glu6	4.5	4.34
Glu13	4.4	4.42		His12	6.2	4.91		Asp10	6.1	6.19
Asp16	4	3.70		Asp14	2	5.44		Glu32	3.6	2.16
Asp20	3.8	3.08		Asp38	3.5	3.44		Glu48	4.4	4.46
Asp26	7.5	7.78		His48	6	8.00		Glu57	3.2	2.72
Glu47	4.1	4.81		Glu49	4.7	3.08		Glu61	3.9	3.73
Glu56	5.1	4.57		Asp53	3.9	3.38		His62	7	6.39
Asp58	5.3	8.17		Asp83	3.5	5.91		Glu64	4.4	3.77
Asp60	2.8	2.96		Glu86	4.1	5.84		Asp70	2.6	6.52
Asp61	4.2	4.82		His105	6.7	5.52		Asp83	5.5	5.70
Asp64	3.2	3.27		Glu111	3.6	4.02		Asp94	3.2	3.05
Glu68	4.9	4.60		His119	6.1	5.64		Asp108	3.2	1.89
Glu70	4.6	4.41		Asp121	3.1	4.46		Glu119	4.1	4.51
Glu88	3.7	3.39						His124	7.1	6.37
Glu95	4.1	3.93						His127	7.9	6.79
Glu98	3.9	4.85						Glu129	3.6	4.51
Glu103	4.4	4.71						Glu131	4.3	5.38
								Asp134	4.1	3.85
								Glu135	4.3	4.50
								Glu147	4.2	4.33
								Glu154	4.4	4.00

**NTL**	**Exp**	**Pred**		
Asp8	3	2.54		
Glu17	3.6	3.49		
Asp23	3.1	3.42		
Glu38	4	4.59		
Glu48	4.2	4.08		
Glu54	4.2	4.43		
**HP36**				
Asp44	3.1	1.89		
Glu45	4	4.14		
Asp46	3.5	3.71		
Glu72	4.4	5.14		
**Overall RMSE:**				
HEWL	1.24			
OMTKY	0.85			
Thioredoxin	0.82			
SNase	1.55			
RNaseA	1.59			
BBL	0.76			
Xylanase	2.38			
RNaseH	1.08			
NTL	0.35			
HP36	0.72			

Table S4. Table of pK_a values for benchmark proteins of residues with available experimental data and predicted pK_a values from ***combining pH-AFED with d-AFED***

**HEWL**	**Exp**	**pH-AFED**		**SNase**	**Exp**	**pH-AFED**		**BBL**	**Exp**	**pH-AFED**
Glu7	2.6	3.36		His8	6.5	6.13		Asp129	3.9	3.43
His15	5.5	5.19		Glu10	2.8	3.65		Glu141	4.5	4.37
Asp18	2.8	3.19		Asp19	2.2	2.62		His142	6.5	6.03
Glu35	6.1	5.79		Asp21	6.5	3.96		Asp145	3.7	3.23
Asp48	1.4	2.30		Asp40	3.9	3.56		Glu161	3.7	4.03
Asp52	3.6	4.85		Glu43	4.3	3.91		Asp162	3.2	2.31
Asp66	1.2	2.52		Glu52	3.9	4.34		Glu164	4.5	3.94
Asp87	2.2	2.49		Glu57	3.5	4.41		His166	5.4	5.56
Asp101	4.5	4.69		Glu67	3.8	4.32				
Asp119	3.5	3.02		Glu73	3.3	3.92				
				Glu75	3.3	4.39		**Xylanase**		
**OMTKY**				Asp95	2.2	3.69		Asp5	3	3.61
Asp8	2.7	2.83		Glu101	3.8	4.11		Asp12	2.5	3.90
Glu11	4.1	3.74		His121	5.2	5.32		Glu79	4.6	5.54
Glu20	3.2	3.66		Glu122	3.9	4.25		Asp107	2.7	3.32
Asp27	2.3	3.17		Glu129	3.8	3.62		Asp120	3.2	3.27
Glu44	4.8	4.20		Glu135	3.8	3.36		Asp122	3.6	3.82
His53	7.5	6.66						His157	6.5	5.87
				**RNaseA**						
**Thioredoxin**				Glu2	2.8	3.35		**RNaseH**		
Glu6	4.8	4.45		Glu9	4	4.67		Glu6	4.5	4.42
Glu13	4.4	4.32		His12	6.2	5.26		Asp10	6.1	5.03
Asp16	4	3.80		Asp14	2	3.75		Glu32	3.6	3.28
Asp20	3.8	3.08		Asp38	3.5	3.33		Glu48	4.4	4.51
Asp26	7.5	7.23		His48	6	5.53		Glu57	3.2	3.74
Glu47	4.1	4.37		Glu49	4.7	4.32		Glu61	3.9	3.79
Glu56	5.1	4.64		Asp53	3.9	3.36		His62	7	6.07
Asp58	5.3	4.82		Asp83	3.5	4.93		Glu64	4.4	4.11
Asp60	2.8	3.25		Glu86	4.1	4.84		Asp70	2.6	4.42
Asp61	4.2	4.28		His105	6.7	5.78		Asp83	5.5	5.70
Asp64	3.2	2.88		Glu111	3.6	4.18		Asp94	3.2	3.17
Glu68	4.9	4.40		His119	6.1	5.79		Asp108	3.2	3.14
Glu70	4.6	4.22		Asp121	3.1	3.80		Glu119	4.1	4.21
Glu88	3.7	3.59						His124	7.1	6.29
Glu95	4.1	4.05						His127	7.9	6.24
Glu98	3.9	4.38						Glu129	3.6	4.23
Glu103	4.4	4.34						Glu131	4.3	4.24
								Asp134	4.1	3.84
								Glu135	4.3	4.21
								Glu147	4.2	4.12
								Glu154	4.4	4.09

**NTL**	**Exp**	**pH-AFED**		
Asp8	3	2.85		
Glu17	3.6	3.63		
Asp23	3.1	3.16		
Glu38	4	4.17		
Glu48	4.2	3.98		
Glu54	4.2	4.17		
**HP36**				
Asp44	3.1	2.69		
Glu45	4	3.98		
Asp46	3.5	3.46		
Glu72	4.4	4.58		
**Overall RMSE:**				
HEWL	0.74			
OMTKY	0.36			
Thioredoxin	0.36			
SNase	0.86			
RNaseA	0.84			
BBL	0.49			
Xylanase	0.76			
RNaseH	0.69			
NTL	0.13			
HP36	0.22			

Quantifying Mixed States Using pH-AFED

$$\Delta G_{pH-AFED} = -k_b T_\lambda \ln(P_\lambda)$$

Starting from Eq. 6 in the main text, the following can be obtained as the free energy contributions of mixed states vs non-mixed states comparing those sampled from a pH-AFED simulation and those expected from a standard MD simulation at 300 K:

$$\Delta G_{mixed-non\_mixed} = -k_b T_\lambda \ln\left(\frac{n_{mixed-AFED}}{n_{non-mixed-AFED}}\right) = -k_b T \ln\left(\frac{n_{mixed}}{n_{non-mixed}}\right)$$

$$\left(\frac{n_{mixed-AFED}}{n_{non-mixed-AFED}}\right)^{\frac{T_\lambda}{300}} = \left(\frac{n_{mixed}}{n_{non-mixed)}}\right) = mixed\ to\ non\ mixed\ ratio$$

After solving for the mixed to non-mixed ratio, one can obtain the mixed fraction:

$$n_{mixed-fraction} = 1 - \left(\frac{1}{1 + \left(\frac{n_{mixed}}{n_{non-mixed}}\right)}\right)$$

Table S5. Table of fractions of mixed lambda states (λ values between 0.2 and 0.8) for parameters used in the main text **(T_λ = 750, $m_\lambda = 750$, Barrier = 10 kJ/mol) in black**, and those from simulations with a larger barrier **height (T_λ = 750, $m_\lambda = 750$, Barrier = 15 kJ/mol) in blue**

HEWL

pH0	pH1	pH2	pH3	pH4	pH5	pH6	pH7	pH8
GLU7	GLU7	GLU7	GLU7	GLU7	GLU7	GLU7	GLU7	GLU7
0.00	0.01	0.07	0.22	0.36	0.22	0.07	0.01	0.00
HIS15	HIS15	HIS15	HIS15	HIS15	HIS15	HIS15	HIS15	HIS15
0.00	0.00	0.01	0.01	0.05	0.12	0.23	0.30	0.25
ASP18	ASP18	ASP18	ASP18	ASP18	ASP18	ASP18	ASP18	ASP18
0.01	0.03	0.22	0.41	0.50	0.17	0.06	0.00	0.00
GLU35	GLU35	GLU35	GLU35	GLU35	GLU35	GLU35	GLU35	GLU35
0.00	0.00	0.01	0.13	0.38	0.52	0.37	0.09	0.11
ASP48	ASP48	ASP48	ASP48	ASP48	ASP48	ASP48	ASP48	ASP48
0.02	0.04	0.21	0.38	0.29	0.15	0.01	0.00	0.00
ASP52	ASP52	ASP52	ASP52	ASP52	ASP52	ASP52	ASP52	ASP52
0.00	0.02	0.11	0.33	0.49	0.35	0.19	0.03	0.01
ASP66	ASP66	ASP66	ASP66	ASP66	ASP66	ASP66	ASP66	ASP66
0.01	0.04	0.47	0.43	0.51	0.12	0.02	0.01	0.00
ASP87	ASP87	ASP87	ASP87	ASP87	ASP87	ASP87	ASP87	ASP87
0.00	0.03	0.15	0.33	0.32	0.05	0.02	0.00	0.00
ASP101	ASP101	ASP101	ASP101	ASP101	ASP101	ASP101	ASP101	ASP101
0.00	0.02	0.09	0.32	0.45	0.30	0.08	0.03	0.00
ASP119	ASP119	ASP119	ASP119	ASP119	ASP119	ASP119	ASP119	ASP119
0.00	0.03	0.14	0.38	0.36	0.17	0.03	0.01	0.00

HEWL

pH0	pH1	pH2	pH3	pH4	pH5	pH6	pH7	pH8
GLU7	GLU7	GLU7	GLU7	GLU7	GLU7	GLU7	GLU7	GLU7
0.00	0.01	0.04	0.11	0.22	0.09	0.02	0.00	0.00
HIS15	HIS15	HIS15	HIS15	HIS15	HIS15	HIS15	HIS15	HIS15
0.00	0.00	0.00	0.00	0.01	0.04	0.08	0.08	0.03
ASP18	ASP18	ASP18	ASP18	ASP18	ASP18	ASP18	ASP18	ASP18
0.00	0.02	0.07	0.19	0.26	0.12	0.02	0.00	0.00
GLU	GLU	GLU	GLU	GLU	GLU	GLU	GLU	GLU
0.00	0.00	0.02	0.06	0.31	0.32	0.27	0.07	0.02
ASP48	ASP48	ASP48	ASP48	ASP48	ASP48	ASP48	ASP48	ASP48
0.00	0.03	0.09	0.12	0.16	0.03	0.00	0.00	0.00
ASP52	ASP52	ASP52	ASP52	ASP52	ASP52	ASP52	ASP52	ASP52

0.00	0.01	0.04	0.18	0.31	0.16	0.04	0.01	0.00
ASP66	ASP66	ASP66	ASP66	ASP66	ASP66	ASP66	ASP66	ASP66
0.01	0.03	0.30	0.35	0.27	0.08	0.01	0.00	0.00
ASP87	ASP87	ASP87	ASP87	ASP87	ASP87	ASP87	ASP87	ASP87
0.00	0.02	0.06	0.18	0.17	0.03	0.01	0.00	0.00
ASP101	ASP101	ASP101	ASP101	ASP101	ASP101	ASP101	ASP101	ASP101
0.00	0.01	0.03	0.17	0.29	0.14	0.03	0.01	0.00
ASP119	ASP119	ASP119	ASP119	ASP119	ASP119	ASP119	ASP119	ASP119
0.00	0.02	0.06	0.19	0.22	0.09	0.01	0.00	0.00

Thioredoxin

pH0	pH1	pH2	pH3	pH4	pH5	pH6	pH7	pH8
GLU6	GLU6	GLU6	GLU6	GLU6	GLU6	GLU6	GLU6	GLU6
0.00	0.01	0.03	0.17	0.37	0.30	0.12	0.02	0.00
GLU13	GLU13	GLU13	GLU13	GLU13	GLU13	GLU13	GLU13	GLU13
0.00	0.01	0.05	0.20	0.34	0.32	0.07	0.03	0.00
ASP16	ASP16	ASP16	ASP16	ASP16	ASP16	ASP16	ASP16	ASP16
0.00	0.02	0.10	0.32	0.37	0.19	0.03	0.01	0.00
ASP20	ASP20	ASP20	ASP20	ASP20	ASP20	ASP20	ASP20	ASP20
0.00	0.03	0.12	0.33	0.36	0.20	0.02	0.00	0.00
ASP26	ASP26	ASP26	ASP26	ASP26	ASP26	ASP26	ASP26	ASP26
0.03	0.07	0.14	0.27	0.28	0.33	0.33	0.25	0.08
GLU47	GLU47	GLU47	GLU47	GLU47	GLU47	GLU47	GLU47	GLU47
0.00	0.01	0.05	0.18	0.41	0.29	0.13	0.02	0.00
GLU47	GLU47	GLU47	GLU47	GLU47	GLU47	GLU47	GLU47	GLU47
0.00	0.02	0.08	0.26	0.51	0.38	0.20	0.03	0.00
ASP58	ASP58	ASP58	ASP58	ASP58	ASP58	ASP58	ASP58	ASP58
0.00	0.03	0.10	0.47	0.51	0.36	0.16	0.03	0.00
ASP60	ASP60	ASP60	ASP60	ASP60	ASP60	ASP60	ASP60	ASP60
0.01	0.02	0.21	0.43	0.45	0.31	0.08	0.02	0.00
ASP61	ASP61	ASP61	ASP61	ASP61	ASP61	ASP61	ASP61	ASP61
0.00	0.03	0.12	0.32	0.39	0.24	0.07	0.01	0.00
ASP64	ASP64	ASP64	ASP64	ASP64	ASP64	ASP64	ASP64	ASP64
0.00	0.04	0.12	0.37	0.36	0.18	0.02	0.00	0.00
GLU68	GLU68	GLU68	GLU68	GLU68	GLU68	GLU68	GLU68	GLU68
0.00	0.01	0.04	0.27	0.40	0.28	0.09	0.01	0.00
GLU70	GLU70	GLU70	GLU70	GLU70	GLU70	GLU70	GLU70	GLU70
0.00	0.01	0.05	0.19	0.36	0.26	0.10	0.02	0.00
GLU88	GLU88	GLU88	GLU88	GLU88	GLU88	GLU88	GLU88	GLU88
0.00	0.01	0.05	0.25	0.34	0.26	0.09	0.01	0.00
GLU95	GLU95	GLU95	GLU95	GLU95	GLU95	GLU95	GLU95	GLU95

0.00	0.01	0.04	0.23	0.38	0.26	0.09	0.02	0.00
GLU98	GLU98	GLU98	GLU98	GLU98	GLU98	GLU98	GLU98	GLU98
0.00	0.01	0.05	0.23	0.41	0.32	0.12	0.03	0.01
GLU103	GLU103	GLU103	GLU103	GLU103	GLU103	GLU103	GLU103	GLU103
0.00	0.00	0.05	0.23	0.40	0.34	0.12	0.02	0.00

Thioredoxin

pH0	pH1	pH2	pH3	pH4	pH5	pH6	pH7	pH8
GLU6	GLU6	GLU6	GLU6	GLU6	GLU6	GLU6	GLU6	GLU6
0.00	0.00	0.01	0.08	0.18	0.13	0.05	0.01	0.00
GLU13	GLU13	GLU13	GLU13	GLU13	GLU13	GLU13	GLU13	GLU13
0.00	0.00	0.01	0.06	0.19	0.16	0.05	0.01	0.00
ASP16	ASP16	ASP16	ASP16	ASP16	ASP16	ASP16	ASP16	ASP16
0.00	0.01	0.05	0.19	0.24	0.07	0.02	0.00	0.00
ASP20	ASP20	ASP20	ASP20	ASP20	ASP20	ASP20	ASP20	ASP20
0.00	0.02	0.06	0.15	0.20	0.05	0.01	0.00	0.00
ASP26	ASP26	ASP26	ASP26	ASP26	ASP26	ASP26	ASP26	ASP26
0.03	0.10	0.21	0.25	0.32	0.35	0.25	0.16	0.09
GLU47	GLU47	GLU47	GLU47	GLU47	GLU47	GLU47	GLU47	GLU47
0.00	0.00	0.02	0.10	0.21	0.17	0.05	0.01	0.00
GLU47	GLU47	GLU47	GLU47	GLU47	GLU47	GLU47	GLU47	GLU47
0.00	0.01	0.03	0.09	0.30	0.19	0.06	0.01	0.00
ASP58	ASP58	ASP58	ASP58	ASP58	ASP58	ASP58	ASP58	ASP58
0.00	0.00	0.05	0.19	0.30	0.12	0.03	0.01	0.00
ASP60	ASP60	ASP60	ASP60	ASP60	ASP60	ASP60	ASP60	ASP60
0.00	0.02	0.06	0.19	0.29	0.12	0.04	0.00	0.00
ASP61	ASP61	ASP61	ASP61	ASP61	ASP61	ASP61	ASP61	ASP61
0.00	0.01	0.06	0.15	0.24	0.12	0.03	0.00	0.00
ASP64	ASP64	ASP64	ASP64	ASP64	ASP64	ASP64	ASP64	ASP64
0.00	0.02	0.06	0.17	0.16	0.05	0.01	0.00	0.00
GLU68	GLU68	GLU68	GLU68	GLU68	GLU68	GLU68	GLU68	GLU68
0.00	0.00	0.02	0.08	0.22	0.15	0.04	0.01	0.00
GLU70	GLU70	GLU70	GLU70	GLU70	GLU70	GLU70	GLU70	GLU70
0.00	0.00	0.02	0.07	0.19	0.12	0.04	0.01	0.00
GLU88	GLU88	GLU88	GLU88	GLU88	GLU88	GLU88	GLU88	GLU88
0.00	0.00	0.03	0.10	0.18	0.09	0.04	0.01	0.00
GLU95	GLU95	GLU95	GLU95	GLU95	GLU95	GLU95	GLU95	GLU95
0.00	0.00	0.02	0.10	0.20	0.16	0.03	0.01	0.00
GLU98	GLU98	GLU98	GLU98	GLU98	GLU98	GLU98	GLU98	GLU98
0.00	0.00	0.02	0.08	0.24	0.14	0.05	0.01	0.00
GLU103	GLU103	GLU103	GLU103	GLU103	GLU103	GLU103	GLU103	GLU103

0.00	0.00	0.02	0.08	0.25	0.14	0.05	0.01	0.00

SNase

pH0	pH1	pH2	pH3	pH4	pH5	pH6	pH7	pH8
HIS8	HIS8	HIS8	HIS8	HIS8	HIS8	HIS8	HIS8	HIS8
0.00	0.00	0.00	0.01	0.02	0.12	0.23	0.33	0.24
GLU47	GLU47	GLU47	GLU47	GLU47	GLU47	GLU47	GLU47	GLU47
0.00	0.01	0.06	0.20	0.39	0.31	0.11	0.01	0.00
ASP19	ASP19	ASP19	ASP19	ASP19	ASP19	ASP19	ASP19	ASP19
0.00	0.03	0.14	0.30	0.28	0.09	0.02	0.00	0.00
ASP21	ASP21	ASP21	ASP21	ASP21	ASP21	ASP21	ASP21	ASP21
0.07	0.12	0.18	0.26	0.25	0.17	0.12	0.07	0.04
ASP40	ASP40	ASP40	ASP40	ASP40	ASP40	ASP40	ASP40	ASP40
0.00	0.03	0.16	0.33	0.38	0.22	0.03	0.01	0.00
GLU43	GLU43	GLU43	GLU43	GLU43	GLU43	GLU43	GLU43	GLU43
0.00	0.01	0.05	0.18	0.35	0.31	0.08	0.02	0.00
GLU52	GLU52	GLU52	GLU52	GLU52	GLU52	GLU52	GLU52	GLU52
0.00	0.01	0.05	0.21	0.40	0.36	0.12	0.03	0.00
GLU57	GLU57	GLU57	GLU57	GLU57	GLU57	GLU57	GLU57	GLU57
0.00	0.01	0.05	0.31	0.46	0.35	0.13	0.03	0.00
GLU67	GLU67	GLU67	GLU67	GLU67	GLU67	GLU67	GLU67	GLU67
0.00	0.01	0.06	0.26	0.52	0.43	0.17	0.03	0.00
GLU73	GLU73	GLU73	GLU73	GLU73	GLU73	GLU73	GLU73	GLU73
0.00	0.01	0.06	0.27	0.43	0.33	0.12	0.02	0.00
GLU75	GLU75	GLU75	GLU75	GLU75	GLU75	GLU75	GLU75	GLU75
0.04	0.08	0.13	0.19	0.28	0.23	0.16	0.12	0.06
ASP95	ASP95	ASP95	ASP95	ASP95	ASP95	ASP95	ASP95	ASP95
0.00	0.04	0.20	0.48	0.53	0.32	0.08	0.03	0.00
GLU101	GLU101	GLU101	GLU101	GLU101	GLU101	GLU101	GLU101	GLU101
0.00	0.02	0.06	0.30	0.48	0.39	0.19	0.03	0.00
HIS121	HIS121	HIS121	HIS121	HIS121	HIS121	HIS121	HIS121	HIS121
0.00	0.00	0.00	0.01	0.03	0.09	0.26	0.32	0.15
GLU122	GLU122	GLU122	GLU122	GLU122	GLU122	GLU122	GLU122	GLU122
0.00	0.01	0.03	0.22	0.42	0.44	0.24	0.04	0.00
GLU129	GLU129	GLU129	GLU129	GLU129	GLU129	GLU129	GLU129	GLU129
0.00	0.03	0.08	0.36	0.58	0.45	0.21	0.04	0.01
GLU135	GLU135	GLU135	GLU135	GLU135	GLU135	GLU135	GLU135	GLU135
0.00	0.01	0.09	0.21	0.33	0.26	0.06	0.01	0.00

SNase

pH0	pH1	pH2	pH3	pH4	pH5	pH6	pH7	pH8
HIS8	HIS8	HIS8	HIS8	HIS8	HIS8	HIS8	HIS8	HIS8
0.00	0.00	0.00	0.00	0.00	0.00	0.00	0.01	0.00
GLU47	GLU47	GLU47	GLU47	GLU47	GLU47	GLU47	GLU47	GLU47
0.00	0.00	0.00	0.01	0.07	0.02	0.00	0.00	0.00
ASP19	ASP19	ASP19	ASP19	ASP19	ASP19	ASP19	ASP19	ASP19
0.00	0.00	0.00	0.05	0.03	0.00	0.00	0.00	0.00
ASP21	ASP21	ASP21	ASP21	ASP21	ASP21	ASP21	ASP21	ASP21
0.01	0.02	0.04	0.12	0.10	0.05	0.02	0.01	0.00
ASP40	ASP40	ASP40	ASP40	ASP40	ASP40	ASP40	ASP40	ASP40
0.00	0.00	0.00	0.05	0.07	0.01	0.00	0.00	0.00
GLU43	GLU43	GLU43	GLU43	GLU43	GLU43	GLU43	GLU43	GLU43
0.00	0.00	0.00	0.01	0.06	0.03	0.00	0.00	0.00
GLU52	GLU52	GLU52	GLU52	GLU52	GLU52	GLU52	GLU52	GLU52
0.00	0.00	0.00	0.01	0.06	0.04	0.00	0.00	0.00
GLU57	GLU57	GLU57	GLU57	GLU57	GLU57	GLU57	GLU57	GLU57
0.00	0.00	0.00	0.01	0.10	0.06	0.01	0.00	0.00
GLU67	GLU67	GLU67	GLU67	GLU67	GLU67	GLU67	GLU67	GLU67
0.00	0.00	0.00	0.01	0.08	0.05	0.00	0.00	0.00
GLU73	GLU73	GLU73	GLU73	GLU73	GLU73	GLU73	GLU73	GLU73
0.00	0.00	0.00	0.01	0.13	0.04	0.00	0.00	0.00
GLU75	GLU75	GLU75	GLU75	GLU75	GLU75	GLU75	GLU75	GLU75
0.00	0.01	0.02	0.07	0.15	0.10	0.04	0.02	0.00
ASP77	ASP77	ASP77	ASP77	ASP77	ASP77	ASP77	ASP77	ASP77
0.01	0.02	0.06	0.10	0.11	0.04	0.01	0.00	0.00
ASP83	ASP83	ASP83	ASP83	ASP83	ASP83	ASP83	ASP83	ASP83
0.00	0.00	0.01	0.20	0.38	0.00	0.00	0.00	0.00
ASP95	ASP95	ASP95	ASP95	ASP95	ASP95	ASP95	ASP95	ASP95
0.00	0.00	0.02	0.09	0.18	0.04	0.00	0.00	0.00
GLU101	GLU101	GLU101	GLU101	GLU101	GLU101	GLU101	GLU101	GLU101
0.00	0.00	0.00	0.01	0.11	0.08	0.00	0.00	0.00
HIS121	HIS121	HIS121	HIS121	HIS121	HIS121	HIS121	HIS121	HIS121
0.00	0.00	0.00	0.00	0.00	0.00	0.00	0.01	0.00
GLU122	GLU122	GLU122	GLU122	GLU122	GLU122	GLU122	GLU122	GLU122
0.00	0.00	0.00	0.01	0.22	0.06	0.00	0.00	0.00
GLU129	GLU129	GLU129	GLU129	GLU129	GLU129	GLU129	GLU129	GLU129
0.00	0.00	0.00	0.04	0.31	0.25	0.01	0.00	0.00
GLU135	GLU135	GLU135	GLU135	GLU135	GLU135	GLU135	GLU135	GLU135
0.00	0.00	0.00	0.01	0.08	0.01	0.00	0.00	0.00

RNaseA

pH0	pH1	pH2	pH3	pH4	pH5	pH6	pH7	pH8
GLU2	GLU2	GLU2	GLU2	GLU2	GLU2	GLU2	GLU2	GLU2
0.00	0.00	0.01	0.06	0.25	0.16	0.00	0.00	0.00
GLU9	GLU9	GLU9	GLU9	GLU9	GLU9	GLU9	GLU9	GLU9
0.00	0.00	0.00	0.08	0.45	0.28	0.07	0.00	0.00
HIS12	HIS12	HIS12	HIS12	HIS12	HIS12	HIS12	HIS12	HIS12
0.00	0.00	0.00	0.00	0.00	0.02	0.10	0.41	0.12
ASP14	ASP14	ASP14	ASP14	ASP14	ASP14	ASP14	ASP14	ASP14
0.00	0.02	0.04	0.22	0.24	0.08	0.01	0.01	0.00
ASP38	ASP38	ASP38	ASP38	ASP38	ASP38	ASP38	ASP38	ASP38
0.00	0.00	0.03	0.21	0.24	0.03	0.00	0.00	0.00
HIS	HIS	HIS	HIS	HIS	HIS	HIS	HIS	HIS
0.00	0.00	0.00	0.00	0.00	0.01	0.04	0.31	0.12
GLU49	GLU49	GLU49	GLU49	GLU49	GLU49	GLU49	GLU49	GLU49
0.00	0.00	0.01	0.09	0.36	0.18	0.02	0.00	0.00
ASP53	ASP53	ASP53	ASP53	ASP53	ASP53	ASP53	ASP53	ASP53
0.00	0.00	0.02	0.18	0.24	0.04	0.00	0.00	0.00
ASP83	ASP83	ASP83	ASP83	ASP83	ASP83	ASP83	ASP83	ASP83
0.00	0.00	0.03	0.47	0.68	0.38	0.06	0.00	0.00
GLU86	GLU86	GLU86	GLU86	GLU86	GLU86	GLU86	GLU86	GLU86
0.00	0.00	0.00	0.05	0.56	0.22	0.09	0.00	0.00
HIS	HIS	HIS	HIS	HIS	HIS	HIS	HIS	HIS
0.00	0.00	0.00	0.00	0.00	0.04	0.13	0.27	0.13
GLU111	GLU111	GLU111	GLU111	GLU111	GLU111	GLU111	GLU111	GLU111
0.00	0.00	0.00	0.11	0.31	0.15	0.04	0.00	0.00
HIS	HIS	HIS	HIS	HIS	HIS	HIS	HIS	HIS
0.00	0.00	0.00	0.00	0.00	0.02	0.05	0.08	0.05
ASP121	ASP121	ASP121	ASP121	ASP121	ASP121	ASP121	ASP121	ASP121
0.00	0.01	0.21	0.80	0.77	0.60	0.32	0.00	0.00

RNaseA

pH0	pH1	pH2	pH3	pH4	pH5	pH6	pH7	pH8
GLU2	GLU2	GLU2	GLU2	GLU2	GLU2	GLU2	GLU2	GLU2
0.00	0.00	0.00	0.03	0.05	0.02	0.00	0.00	0.00
GLU9	GLU9	GLU9	GLU9	GLU9	GLU9	GLU9	GLU9	GLU9
0.00	0.00	0.00	0.01	0.12	0.06	0.01	0.00	0.00
HIS12	HIS12	HIS12	HIS12	HIS12	HIS12	HIS12	HIS12	HIS12

0.00	0.00	0.00	0.00	0.00	0.00	0.02	0.05	0.01
ASP14	ASP14	ASP14	ASP14	ASP14	ASP14	ASP14	ASP14	ASP14
0.01	0.01	0.07	0.22	0.17	0.11	0.03	0.01	0.00
ASP38	ASP38	ASP38	ASP38	ASP38	ASP38	ASP38	ASP38	ASP38
0.00	0.00	0.00	0.02	0.06	0.00	0.00	0.00	0.00
HIS	HIS	HIS	HIS	HIS	HIS	HIS	HIS	HIS
0.00	0.00	0.00	0.00	0.00	0.00	0.00	0.01	0.00
GLU49	GLU49	GLU49	GLU49	GLU49	GLU49	GLU49	GLU49	GLU49
0.00	0.00	0.00	0.02	0.07	0.03	0.00	0.00	0.00
ASP53	ASP53	ASP53	ASP53	ASP53	ASP53	ASP53	ASP53	ASP53
0.00	0.00	0.00	0.03	0.07	0.00	0.00	0.00	0.00
ASP83	ASP83	ASP83	ASP83	ASP83	ASP83	ASP83	ASP83	ASP83
0.00	0.00	0.00	0.10	0.39	0.08	0.01	0.00	0.00
GLU86	GLU86	GLU86	GLU86	GLU86	GLU86	GLU86	GLU86	GLU86
0.00	0.00	0.00	0.01	0.06	0.11	0.00	0.00	0.00
HIS	HIS	HIS	HIS	HIS	HIS	HIS	HIS	HIS
0.00	0.00	0.00	0.00	0.00	0.00	0.02	0.03	0.00
GLU111	GLU111	GLU111	GLU111	GLU111	GLU111	GLU111	GLU111	GLU111
0.00	0.00	0.00	0.01	0.11	0.05	0.00	0.00	0.00
HIS	HIS	HIS	HIS	HIS	HIS	HIS	HIS	HIS
0.00	0.00	0.00	0.00	0.00	0.00	0.00	0.01	0.00
ASP121	ASP121	ASP121	ASP121	ASP121	ASP121	ASP121	ASP121	ASP121
0.00	0.00	0.04	0.23	0.31	0.16	0.01	0.00	0.00

Note: Simulations for ASP121 here were conducted using a barrier height of 20 kJ/mol

Xylanase

pH0	pH1	pH2	pH3	pH4	pH5	pH6	pH7	pH8
ASP5	ASP5	ASP5	ASP5	ASP5	ASP5	ASP5	ASP5	ASP5
0.00	0.02	0.09	0.31	0.39	0.30	0.08	0.02	0.00
ASP12	ASP12	ASP12	ASP12	ASP12	ASP12	ASP12	ASP12	ASP12
0.00	0.04	0.12	0.38	0.48	0.29	0.06	0.01	0.00
GLU79	GLU79	GLU79	GLU79	GLU79	GLU79	GLU79	GLU79	GLU79
0.04	0.06	0.16	0.19	0.38	0.35	0.30	0.18	0.06
ASP107	ASP107	ASP107	ASP107	ASP107	ASP107	ASP107	ASP107	ASP107
0.00	0.06	0.23	0.48	0.51	0.23	0.06	0.01	0.00
ASP120	ASP120	ASP120	ASP120	ASP120	ASP120	ASP120	ASP120	ASP120
0.00	0.04	0.15	0.34	0.41	0.28	0.02	0.00	0.00
ASP122	ASP122	ASP122	ASP122	ASP122	ASP122	ASP122	ASP122	ASP122
0.00	0.02	0.10	0.33	0.34	0.17	0.03	0.00	0.00

HIS157	HIS157	HIS157	HIS157	HIS157	HIS157	HIS157	HIS157	HIS157
0.00	0.00	0.00	0.01	0.02	0.09	0.20	0.35	0.25

Xylanase

pH0	pH1	pH2	pH3	pH4	pH5	pH6	pH7	pH8
ASP5	ASP5	ASP5	ASP5	ASP5	ASP5	ASP5	ASP5	ASP5
0.00	0.01	0.03	0.20	0.24	0.10	0.03	0.00	0.00
ASP12	ASP12	ASP12	ASP12	ASP12	ASP12	ASP12	ASP12	ASP12
0.00	0.01	0.08	0.21	0.31	0.07	0.02	0.01	0.00
GLU79	GLU79	GLU79	GLU79	GLU79	GLU79	GLU79	GLU79	GLU79
0.02	0.07	0.15	0.16	0.33	0.33	0.21	0.14	0.14
ASP107	ASP107	ASP107	ASP107	ASP107	ASP107	ASP107	ASP107	ASP107
0.00	0.02	0.10	0.25	0.31	0.14	0.02	0.00	0.00
ASP120	ASP120	ASP120	ASP120	ASP120	ASP120	ASP120	ASP120	ASP120
0.00	0.02	0.05	0.18	0.23	0.11	0.01	0.00	0.00
ASP122	ASP122	ASP122	ASP122	ASP122	ASP122	ASP122	ASP122	ASP122
0.00	0.01	0.05	0.17	0.19	0.06	0.02	0.00	0.00
HIS157	HIS157	HIS157	HIS157	HIS157	HIS157	HIS157	HIS157	HIS157
0.00	0.00	0.00	0.00	0.01	0.02	0.06	0.15	0.05

RNaseH

pH0	pH1	pH2	pH3	pH4	pH5	pH6	pH7	pH8
GLU6	GLU6	GLU6	GLU6	GLU6	GLU6	GLU6	GLU6	GLU6
0.00	0.01	0.09	0.21	0.40	0.37	0.09	0.02	0.00
ASP10	ASP10	ASP10	ASP10	ASP10	ASP10	ASP10	ASP10	ASP10
0.06	0.10	0.18	0.25	0.28	0.23	0.14	0.07	0.03
GLU32	GLU32	GLU32	GLU32	GLU32	GLU32	GLU32	GLU32	GLU32
0.00	0.02	0.06	0.20	0.21	0.05	0.03	0.00	0.00
GLU48	GLU48	GLU48	GLU48	GLU48	GLU48	GLU48	GLU48	GLU48
0.04	0.09	0.14	0.22	0.32	0.25	0.22	0.11	0.05
GLU57	GLU57	GLU57	GLU57	GLU57	GLU57	GLU57	GLU57	GLU57
0.00	0.02	0.04	0.26	0.37	0.18	0.04	0.00	0.00
GLU61	GLU61	GLU61	GLU61	GLU61	GLU61	GLU61	GLU61	GLU61
0.00	0.01	0.06	0.25	0.48	0.26	0.25	0.01	0.00
HIS62	HIS62	HIS62	HIS62	HIS62	HIS62	HIS62	HIS62	HIS62
0.00	0.00	0.00	0.01	0.02	0.07	0.17	0.28	0.17
GLU64	GLU64	GLU64	GLU64	GLU64	GLU64	GLU64	GLU64	GLU64
0.00	0.00	0.05	0.19	0.42	0.41	0.13	0.02	0.00
ASP70	ASP70	ASP70	ASP70	ASP70	ASP70	ASP70	ASP70	ASP70

0.07	0.09	0.16	0.21	0.26	0.22	0.13	0.09	0.05
HIS83	HIS83	HIS83	HIS83	HIS83	HIS83	HIS83	HIS83	HIS83
0.00	0.00	0.00	0.01	0.02	0.07	0.19	0.29	0.16
ASP94	ASP94	ASP94	ASP94	ASP94	ASP94	ASP94	ASP94	ASP94
0.01	0.03	0.12	0.34	0.35	0.14	0.03	0.01	0.00
ASP102	ASP102	ASP102	ASP102	ASP102	ASP102	ASP102	ASP102	ASP102
0.06	0.10	0.17	0.22	0.24	0.16	0.13	0.07	0.04
ASP108	ASP108	ASP108	ASP108	ASP108	ASP108	ASP108	ASP108	ASP108
0.07	0.12	0.18	0.25	0.23	0.20	0.10	0.08	0.04
GLU119	GLU119	GLU119	GLU119	GLU119	GLU119	GLU119	GLU119	GLU119
0.00	0.01	0.04	0.19	0.41	0.32	0.13	0.03	0.00
HIS124	HIS124	HIS124	HIS124	HIS124	HIS124	HIS124	HIS124	HIS124
0.00	0.00	0.00	0.00	0.02	0.07	0.17	0.24	0.14
HIS127	HIS127	HIS127	HIS127	HIS127	HIS127	HIS127	HIS127	HIS127
0.00	0.00	0.00	0.00	0.04	0.08	0.21	0.33	0.20
GLU129	GLU129	GLU129	GLU129	GLU129	GLU129	GLU129	GLU129	GLU129
0.04	0.07	0.14	0.20	0.29	0.25	0.17	0.12	0.06
GLU131	GLU131	GLU131	GLU131	GLU131	GLU131	GLU131	GLU131	GLU131
0.00	0.01	0.03	0.26	0.41	0.32	0.17	0.03	0.00
ASP134	ASP134	ASP134	ASP134	ASP134	ASP134	ASP134	ASP134	ASP134
0.07	0.11	0.16	0.23	0.23	0.19	0.13	0.07	0.04
GLU135	GLU135	GLU135	GLU135	GLU135	GLU135	GLU135	GLU135	GLU135
0.00	0.00	0.04	0.15	0.38	0.32	0.13	0.02	0.00
GLU147	GLU147	GLU147	GLU147	GLU147	GLU147	GLU147	GLU147	GLU147
0.00	0.01	0.06	0.19	0.37	0.29	0.09	0.02	0.00
GLU154	GLU154	GLU154	GLU154	GLU154	GLU154	GLU154	GLU154	GLU154
0.00	0.01	0.07	0.20	0.37	0.29	0.09	0.02	0.00

RNaseH

pH0	pH1	pH2	pH3	pH4	pH5	pH6	pH7	pH8
GLU6	GLU6	GLU6	GLU6	GLU6	GLU6	GLU6	GLU6	GLU6
0.00	0.00	0.02	0.13	0.30	0.25	0.03	0.01	0.00
ASP10	ASP10	ASP10	ASP10	ASP10	ASP10	ASP10	ASP10	ASP10
0.07	0.13	0.16	0.27	0.27	0.23	0.14	0.08	0.05
GLU32	GLU32	GLU32	GLU32	GLU32	GLU32	GLU32	GLU32	GLU32
0.00	0.01	0.02	0.04	0.08	0.01	0.01	0.00	0.00
GLU48	GLU48	GLU48	GLU48	GLU48	GLU48	GLU48	GLU48	GLU48
0.05	0.09	0.13	0.21	0.28	0.24	0.20	0.12	0.06
GLU57	GLU57	GLU57	GLU57	GLU57	GLU57	GLU57	GLU57	GLU57
0.00	0.01	0.04	0.14	0.22	0.08	0.05	0.00	0.00

GLU61	GLU61	GLU61	GLU61	GLU61	GLU61	GLU61	GLU61	GLU61
0.00	0.00	0.03	0.07	0.21	0.29	0.04	0.01	0.00
HIS	HIS	HIS	HIS	HIS	HIS	HIS	HIS	HIS
0.00	0.00	0.00	0.00	0.01	0.01	0.04	0.08	0.04
GLU64	GLU64	GLU64	GLU64	GLU64	GLU64	GLU64	GLU64	GLU64
0.00	0.01	0.03	0.09	0.24	0.18	0.06	0.01	0.00
ASP70	ASP70	ASP70	ASP70	ASP70	ASP70	ASP70	ASP70	ASP70
0.06	0.10	0.17	0.23	0.26	0.20	0.11	0.10	0.06
HIS83	HIS83	HIS83	HIS83	HIS83	HIS83	HIS83	HIS83	HIS83
0.00	0.00	0.00	0.00	0.01	0.02	0.04	0.07	0.02
ASP94	ASP94	ASP94	ASP94	ASP94	ASP94	ASP94	ASP94	ASP94
0.00	0.01	0.05	0.17	0.17	0.08	0.01	0.00	0.00
ASP102	ASP102	ASP102	ASP102	ASP102	ASP102	ASP102	ASP102	ASP102
0.05	0.09	0.17	0.22	0.24	0.17	0.13	0.06	0.04
ASP108	ASP108	ASP108	ASP108	ASP108	ASP108	ASP108	ASP108	ASP108
0.06	0.09	0.19	0.25	0.26	0.17	0.11	0.07	0.03
GLU119	GLU119	GLU119	GLU119	GLU119	GLU119	GLU119	GLU119	GLU119
0.00	0.00	0.01	0.09	0.21	0.14	0.05	0.01	0.00
HIS124	HIS124	HIS124	HIS124	HIS124	HIS124	HIS124	HIS124	HIS124
0.00	0.00	0.00	0.00	0.00	0.01	0.05	0.08	0.04
HIS127	HIS127	HIS127	HIS127	HIS127	HIS127	HIS127	HIS127	HIS127
0.00	0.00	0.00	0.00	0.00	0.01	0.05	0.09	0.05
GLU129	GLU129	GLU129	GLU129	GLU129	GLU129	GLU129	GLU129	GLU129
0.04	0.07	0.13	0.19	0.28	0.23	0.17	0.11	0.06
GLU131	GLU131	GLU131	GLU131	GLU131	GLU131	GLU131	GLU131	GLU131
0.00	0.01	0.03	0.12	0.24	0.17	0.05	0.02	0.00
ASP134	ASP134	ASP134	ASP134	ASP134	ASP134	ASP134	ASP134	ASP134
0.07	0.08	0.16	0.21	0.25	0.20	0.12	0.07	0.04
GLU135	GLU135	GLU135	GLU135	GLU135	GLU135	GLU135	GLU135	GLU135
0.00	0.00	0.02	0.08	0.23	0.15	0.05	0.01	0.00
GLU147	GLU147	GLU147	GLU147	GLU147	GLU147	GLU147	GLU147	GLU147
0.00	0.00	0.02	0.07	0.21	0.16	0.03	0.01	0.00
GLU154	GLU154	GLU154	GLU154	GLU154	GLU154	GLU154	GLU154	GLU154
0.00	0.00	0.02	0.09	0.20	0.14	0.04	0.01	0.00

1OMU

pH0	pH1	pH2	pH3	pH4	pH5	pH6	pH7	pH8
ASP8	ASP8	ASP8	ASP8	ASP8	ASP8	ASP8	ASP8	ASP8
0.00	0.04	0.15	0.31	0.38	0.16	0.02	0.00	0.00

GLU11	GLU11	GLU11	GLU11	GLU11	GLU11	GLU11	GLU11	GLU11
0.00	0.01	0.04	0.24	0.35	0.26	0.06	0.01	0.00
GLU20	GLU20	GLU20	GLU20	GLU20	GLU20	GLU20	GLU20	GLU20
0.00	0.01	0.06	0.27	0.28	0.21	0.05	0.01	0.00
ASP28	ASP28	ASP28	ASP28	ASP28	ASP28	ASP28	ASP28	ASP28
0.00	0.02	0.16	0.43	0.39	0.14	0.06	0.01	0.00
GLU44	GLU44	GLU44	GLU44	GLU44	GLU44	GLU44	GLU44	GLU44
0.00	0.01	0.05	0.20	0.40	0.32	0.11	0.02	0.00
HIS53	HIS53	HIS53	HIS53	HIS53	HIS53	HIS53	HIS53	HIS53
0.00	0.00	0.00	0.01	0.02	0.08	0.21	0.31	0.16

1OMU

pH0	pH1	pH2	pH3	pH4	pH5	pH6	pH7	pH8
ASP8	ASP8	ASP8	ASP8	ASP8	ASP8	ASP8	ASP8	ASP8
0.00	0.01	0.07	0.15	0.19	0.04	0.01	0.00	0.00
GLU11	GLU11	GLU11	GLU11	GLU11	GLU11	GLU11	GLU11	GLU11
0.00	0.01	0.02	0.08	0.19	0.10	0.03	0.01	0.00
GLU20	GLU20	GLU20	GLU20	GLU20	GLU20	GLU20	GLU20	GLU20
0.00	0.00	0.02	0.09	0.17	0.09	0.05	0.00	0.00
ASP28	ASP28	ASP28	ASP28	ASP28	ASP28	ASP28	ASP28	ASP28
0.00	0.01	0.05	0.20	0.20	0.07	0.02	0.00	0.00
GLU44	GLU44	GLU44	GLU44	GLU44	GLU44	GLU44	GLU44	GLU44
0.00	0.00	0.02	0.08	0.22	0.13	0.05	0.01	0.00
HIS53	HIS53	HIS53	HIS53	HIS53	HIS53	HIS53	HIS53	HIS53
0.00	0.00	0.00	0.00	0.01	0.01	0.07	0.14	0.06

HP36

pH0	pH1	pH2	pH3	pH4	pH5	pH6	pH7	pH8
ASP44	ASP44	ASP44	ASP44	ASP44	ASP44	ASP44	ASP44	ASP44
0.01	0.05	0.18	0.30	0.33	0.09	0.00	0.00	0.00
GLU47	GLU47	GLU47	GLU47	GLU47	GLU47	GLU47	GLU47	GLU47
0.00	0.00	0.03	0.17	0.33	0.24	0.06	0.01	0.00
ASP46	ASP46	ASP46	ASP46	ASP46	ASP46	ASP46	ASP46	ASP46
0.00	0.03	0.12	0.32	0.37	0.16	0.02	0.01	0.00
GLU72	GLU72	GLU72	GLU72	GLU72	GLU72	GLU72	GLU72	GLU72
0.00	0.01	0.04	0.17	0.33	0.30	0.09	0.02	0.00

HP36

pH0	pH1	pH2	pH3	pH4	pH5	pH6	pH7	pH8
ASP44	ASP44	ASP44	ASP44	ASP44	ASP44	ASP44	ASP44	ASP44
0.00	0.01	0.07	0.14	0.16	0.04	0.00	0.00	0.00
GLU47	GLU47	GLU47	GLU47	GLU47	GLU47	GLU47	GLU47	GLU47
0.00	0.01	0.03	0.08	0.19	0.12	0.03	0.01	0.00
ASP46	ASP46	ASP46	ASP46	ASP46	ASP46	ASP46	ASP46	ASP46
0.00	0.01	0.05	0.19	0.20	0.09	0.02	0.00	0.00
GLU72	GLU72	GLU72	GLU72	GLU72	GLU72	GLU72	GLU72	GLU72
0.00	0.00	0.02	0.07	0.21	0.15	0.04	0.01	0.00

BBL

pH0	pH1	pH2	pH3	pH4	pH5	pH6	pH7	pH8
ASP129	ASP129	ASP129	ASP129	ASP129	ASP129	ASP129	ASP129	ASP129
0.01	0.03	0.13	0.33	0.36	0.16	0.02	0.00	0.00
GLU141	GLU141	GLU141	GLU141	GLU141	GLU141	GLU141	GLU141	GLU141
0.00	0.01	0.04	0.19	0.42	0.31	0.15	0.01	0.00
HIS142	HIS142	HIS142	HIS142	HIS142	HIS142	HIS142	HIS142	HIS142
0.00	0.00	0.01	0.01	0.05	0.10	0.24	0.28	0.24
ASP145	ASP145	ASP145	ASP145	ASP145	ASP145	ASP145	ASP145	ASP145
0.01	0.02	0.16	0.34	0.33	0.13	0.03	0.00	0.00
GLU161	GLU161	GLU161	GLU161	GLU161	GLU161	GLU161	GLU161	GLU161
0.00	0.01	0.06	0.21	0.40	0.27	0.10	0.02	0.00
ASP162	ASP162	ASP162	ASP162	ASP162	ASP162	ASP162	ASP162	ASP162
0.01	0.06	0.15	0.41	0.30	0.15	0.01	0.00	0.00
GLU164	GLU164	GLU164	GLU164	GLU164	GLU164	GLU164	GLU164	GLU164
0.00	0.01	0.04	0.18	0.34	0.33	0.12	0.01	0.00
HIS166	HIS166	HIS166	HIS166	HIS166	HIS166	HIS166	HIS166	HIS166
0.00	0.00	0.00	0.02	0.04	0.10	0.24	0.32	0.19

BBL

pH0	pH1	pH2	pH3	pH4	pH5	pH6	pH7	pH8
ASP129	ASP129	ASP129	ASP129	ASP129	ASP129	ASP129	ASP129	ASP129
0.00	0.02	0.06	0.18	0.06	0.09	0.01	0.00	0.00
GLU141	GLU141	GLU141	GLU141	GLU141	GLU141	GLU141	GLU141	GLU141
0.00	0.00	0.02	0.09	0.22	0.13	0.06	0.01	0.00
HIS142	HIS142	HIS142	HIS142	HIS142	HIS142	HIS142	HIS142	HIS142
0.00	0.00	0.00	0.00	0.01	0.03	0.08	0.10	0.04
ASP145	ASP145	ASP145	ASP145	ASP145	ASP145	ASP145	ASP145	ASP145

0.00	0.02	0.07	0.18	0.17	0.07	0.01	0.00	0.00
GLU161	GLU161	GLU161	GLU161	GLU161	GLU161	GLU161	GLU161	GLU161
0.00	0.01	0.02	0.10	0.25	0.14	0.04	0.01	0.00
ASP162	ASP162	ASP162	ASP162	ASP162	ASP162	ASP162	ASP162	ASP162
0.00	0.02	0.07	0.15	0.11	0.05	0.00	0.00	0.00
GLU164	GLU164	GLU164	GLU164	GLU164	GLU164	GLU164	GLU164	GLU164
0.00	0.00	0.04	0.08	0.21	0.16	0.04	0.01	0.00
HIS166	HIS166	HIS166	HIS166	HIS166	HIS166	HIS166	HIS166	HIS166
0.00	0.00	0.00	0.00	0.01	0.03	0.06	0.11	0.04

Table S6. Table of pK_a values for benchmark proteins of residues with available experimental data and predicted pK_a values from ***pH*-AFED with a larger barrier potential**. Here, non-buried residues are simulated using $T_\lambda = 750\text{K}, m_\lambda = 750, V_{\text{barrier}} = 15$ kJ/mol ,and buried residues are simulated using $T_\lambda = 1500\text{K}, m_\lambda = 1500, V_{\text{barrier}} = 22$ kJ/mol

**HEWL**	**Exp**	**Pred**		**SNase**	**Exp**	**Pred**		**BBL**	**Exp**	**Pred**
Glu7	2.6	4.61		His8	6.5	7.38		Asp129	3.9	3.56
His15	5.5	6.56		Glu10	2.8	4.4		Glu141	4.5	4.75
Asp18	2.8	3.95		Asp19	2.2	3.37		His142	6.5	6.39
Glu35	6.1	6.62		Asp21	6.5	4.82		Asp145	3.7	3.87
Asp48	1.4	3.4		Asp40	3.9	4.22		Glu161	3.7	4.65
Asp52	3.6	4.99		Glu43	4.3	4.87		Asp162	3.2	2.79
Asp66	1.2	2.56		Glu52	3.9	4.66		Glu164	4.5	4.39
Asp87	2.2	3.16		Glu57	3.5	4.2		His166	5.4	6.23
Asp101	4.5	4.03		Glu67	3.8	4.42				
Asp119	3.5	3.46		Glu73	3.3	4.72				
				Glu75	3.3	4.3		**Xylanase**		
**OMTKY**				Asp95	2.2	3.11		Asp5	3	2.68
Asp8	2.7	3.79		Glu101	3.8	5.13		Asp12	2.5	3.85
Glu11	4.1	4.57		His121	5.2	6.9		Glu79	4.6	6.64
Glu20	3.2	4.65		Glu122	3.9	4.73		Asp107	2.7	3.65
Asp27	2.3	3.11		Glu129	3.8	3.54		Asp120	3.2	4.01
Glu44	4.8	4.66		Glu135	3.8	3.74		Asp122	3.6	3.95
His53	7.5	6.82						His157	6.5	6.22
				**RNaseA**						
**Thioredoxin**				Glu2	2.8	4.67		**RNaseH**		
Glu6	4.8	6.46		Glu9	4	4.94		Glu6	4.5	4.95
Glu13	4.4	4.67		His12	6.2	4.6		Asp10	6.1	10.57
Asp16	4	4.04		Asp14	2	4.12		Glu32	3.6	4.35
Asp20	3.8	3.48		Asp38	3.5	3.98		Glu48	4.4	6.24
Asp26	7.5	6.8		His48	6	6.99		Glu57	3.2	4.58
Glu47	4.1	4.4		Glu49	4.7	4.18		Glu61	3.9	4.22
Glu56	5.1	4.79		Asp53	3.9	4.65		His62	7	6.53
Asp58	5.3	4		Asp83	3.5	4.43		Glu64	4.4	4.6
Asp60	2.8	4.1		Glu86	4.1	4.29		Asp70	2.6	3.48
Asp61	4.2	4.2		His105	6.7	6.85		Asp83	5.5	6.09
Asp64	3.2	3.43		Glu111	3.6	3.52		Asp94	3.2	3.91
Glu68	4.9	3.98		His119	6.1	5.93		Asp108	3.2	3.82
Glu70	4.6	4.48		Asp121	3.1	3.83		Glu119	4.1	4.73
Glu88	3.7	4.5						His124	7.1	6.4
Glu95	4.1	4.53						His127	7.9	6.37
Glu98	3.9	4.99						Glu129	3.6	4.58
Glu103	4.4	4.56						Glu131	4.3	4.39
								Asp134	4.1	6.42
								Glu135	4.3	4.62
								Glu147	4.2	4.56
								Glu154	4.4	4.65

**NTL**	**Exp**	**Pred**		
Asp8	3	3.57		
Glu17	3.6	3.93		
Asp23	3.1	3.98		
Glu38	4	4.9		
Glu48	4.2	4.55		
Glu54	4.2	4.59		
**HP36**				
Asp44	3.1	2.45		
Glu45	4	4.57		
Asp46	3.5	3.89		
Glu72	4.4	4.52		

Table S7. Table of pK_a values for benchmark proteins (with structures obtained from RCSB) of residues with available experimental data and predicted pK_a values ***from PROPKA3***

**HEWL**	**Exp**	**Pred**		**SNase**	**Exp**	**Pred**		**BBL**	**Exp**	**Pred**
Glu7	2.6	3.97		His8	6.5	5.71		Asp129	3.9	3.31
His15	5.5	6.36		Glu10	2.8	3.93		Glu141	4.5	4.51
Asp18	2.8	3.23		Asp19	2.2	3.65		His142	6.5	7.17
Glu35	6.1	6.51		Asp21	6.5	5.87		Asp145	3.7	3.51
Asp48	1.4	1.81		Asp40	3.9	4.69		Glu161	3.7	4.57
Asp52	3.6	3.87		Glu43	4.3	5.2		Asp162	3.2	2.78
Asp66	1.2	1.73		Glu52	3.9	4.28		Glu164	4.5	4.55
Asp87	2.2	2.07		Glu57	3.5	4.39		His166	5.4	5.9
Asp101	4.5	3.92		Glu67	3.8	3.77				
Asp119	3.5	3.51		Glu73	3.3	4.47				
				Glu75	3.3	4.87		**Xylanase**		
**OMTKY**				Asp95	2.2	3.41		Asp5	3	2.91
Asp8	2.7	3.79		Glu101	3.8	5.23		Asp12	2.5	3.04
Glu11	4.1	4.57		His121	5.2	5.71		Glu79	4.6	5.81
Glu20	3.2	4.65		Glu122	3.9	4.04		Asp107	2.7	2.55
Asp27	2.3	3.11		Glu129	3.8	3.91		Asp120	3.2	3.27
Glu44	4.8	4.66		Glu135	3.8	3.49		Asp122	3.6	3.93
His53	7.5	6.82						His157	6.5	6.5
				**RNaseA**						
**Thioredoxin**				Glu2	2.8	3.4		**RNaseH**		
Glu6	4.8	5.71		Glu9	4	4.99		Glu6	4.5	4.38
Glu13	4.4	4.74		His12	6.2	3.88		Asp10	6.1	7.35
Asp16	4	3.95		Asp14	2	2.54		Glu32	3.6	3.74
Asp20	3.8	3.31		Asp38	3.5	3.07		Glu48	4.4	6.55
Asp26	7.5	7.41		His48	6	5.88		Glu57	3.2	3.86
Glu47	4.1	4.35		Glu49	4.7	4.71		Glu61	3.9	3.88
Glu56	5.1	5		Asp53	3.9	3.98		His62	7	7.08
Asp58	5.3	3.47		Asp83	3.5	4.38		Glu64	4.4	4.55
Asp60	2.8	5.01		Glu86	4.1	4.27		Asp70	2.6	4.81
Asp61	4.2	4		His105	6.7	6.66		Asp83	5.5	6.08
Asp64	3.2	3.56		Glu111	3.6	4.73		Asp94	3.2	3.03
Glu68	4.9	4.66		His119	6.1	6.4		Asp108	3.2	3.92
Glu70	4.6	4.37		Asp121	3.1	4.04		Glu119	4.1	3.75
Glu88	3.7	3.59						His124	7.1	6.31
Glu95	4.1	4.68						His127	7.9	7.23
Glu98	3.9	3.06						Glu129	3.6	3.56
Glu103	4.4	4.69						Glu131	4.3	4.75
								Asp134	4.1	3.51
								Glu135	4.3	4.53
								Glu147	4.2	4.66
								Glu154	4.4	4.46

**NTL**	**Exp**	**Pred**		
Asp8	3	3.81		
Glu17	3.6	4.44		
Asp23	3.1	2.97		
Glu38	4	4.71		
Glu48	4.2	4.61		
Glu54	4.2	4.57		
**HP36**				
Asp44	3.1	3.53		
Glu45	4	4.62		
Asp46	3.5	3.24		
Glu72	4.4	4.6		

Table S8. Table of pK_a values for benchmark proteins (<u>using 2 ns MD equilibrated structures</u>) of residues with available experimental data and predicted pK_a values ***<u>from PROPKA3</u>***

**HEWL**	**Exp**	**Pred**		**SNase**	**Exp**	**Pred**		**BBL**	**Exp**	**Pred**
Glu7	2.6	4.61		His8	6.5	7.38		Asp129	3.9	3.56
His15	5.5	6.56		Glu10	2.8	4.4		Glu141	4.5	4.75
Asp18	2.8	3.95		Asp19	2.2	3.37		His142	6.5	6.39
Glu35	6.1	6.62		Asp21	6.5	4.82		Asp145	3.7	3.87
Asp48	1.4	3.4		Asp40	3.9	4.22		Glu161	3.7	4.65
Asp52	3.6	4.99		Glu43	4.3	4.87		Asp162	3.2	2.79
Asp66	1.2	2.56		Glu52	3.9	4.66		Glu164	4.5	4.39
Asp87	2.2	3.16		Glu57	3.5	4.2		His166	5.4	6.23
Asp101	4.5	4.03		Glu67	3.8	4.42				
Asp119	3.5	3.46		Glu73	3.3	4.72				
				Glu75	3.3	4.3		**Xylanase**		
**OMTKY**				Asp95	2.2	3.11		Asp5	3	2.68
Asp8	2.7	3.79		Glu101	3.8	5.13		Asp12	2.5	3.85
Glu11	4.1	4.57		His121	5.2	6.9		Glu79	4.6	6.64
Glu20	3.2	4.65		Glu122	3.9	4.73		Asp107	2.7	3.65
Asp27	2.3	3.11		Glu129	3.8	3.54		Asp120	3.2	4.01
Glu44	4.8	4.66		Glu135	3.8	3.74		Asp122	3.6	3.95
His53	7.5	6.82						His157	6.5	6.22
				**RNaseA**						
**Thioredoxin**				Glu2	2.8	4.67		**RNaseH**		
Glu6	4.8	6.46		Glu9	4	4.94		Glu6	4.5	4.95
Glu13	4.4	4.67		His12	6.2	4.6		Asp10	6.1	10.57
Asp16	4	4.04		Asp14	2	4.12		Glu32	3.6	4.35
Asp20	3.8	3.48		Asp38	3.5	3.98		Glu48	4.4	6.24
Asp26	7.5	6.8		His48	6	6.99		Glu57	3.2	4.58
Glu47	4.1	4.4		Glu49	4.7	4.18		Glu61	3.9	4.22
Glu56	5.1	4.79		Asp53	3.9	4.65		His62	7	6.53
Asp58	5.3	4		Asp83	3.5	4.43		Glu64	4.4	4.6
Asp60	2.8	4.1		Glu86	4.1	4.29		Asp70	2.6	3.48
Asp61	4.2	4.2		His105	6.7	6.85		Asp83	5.5	6.09
Asp64	3.2	3.43		Glu111	3.6	3.52		Asp94	3.2	3.91
Glu68	4.9	3.98		His119	6.1	5.93		Asp108	3.2	3.82
Glu70	4.6	4.48		Asp121	3.1	3.83		Glu119	4.1	4.73
Glu88	3.7	4.5						His124	7.1	6.4
Glu95	4.1	4.53						His127	7.9	6.37
Glu98	3.9	4.99						Glu129	3.6	4.58
Glu103	4.4	4.56						Glu131	4.3	4.39
								Asp134	4.1	6.42
								Glu135	4.3	4.62
								Glu147	4.2	4.56
								Glu154	4.4	4.65

**NTL**	**Exp**	**Pred**		
Asp8	3	3.57		
Glu17	3.6	3.93		
Asp23	3.1	3.98		
Glu38	4	4.9		
Glu48	4.2	4.55		
Glu54	4.2	4.59		
**HP36**				
Asp44	3.1	2.45		
Glu45	4	4.57		
Asp46	3.5	3.89		
Glu72	4.4	4.52		

Torsional Analysis for pH-AFED

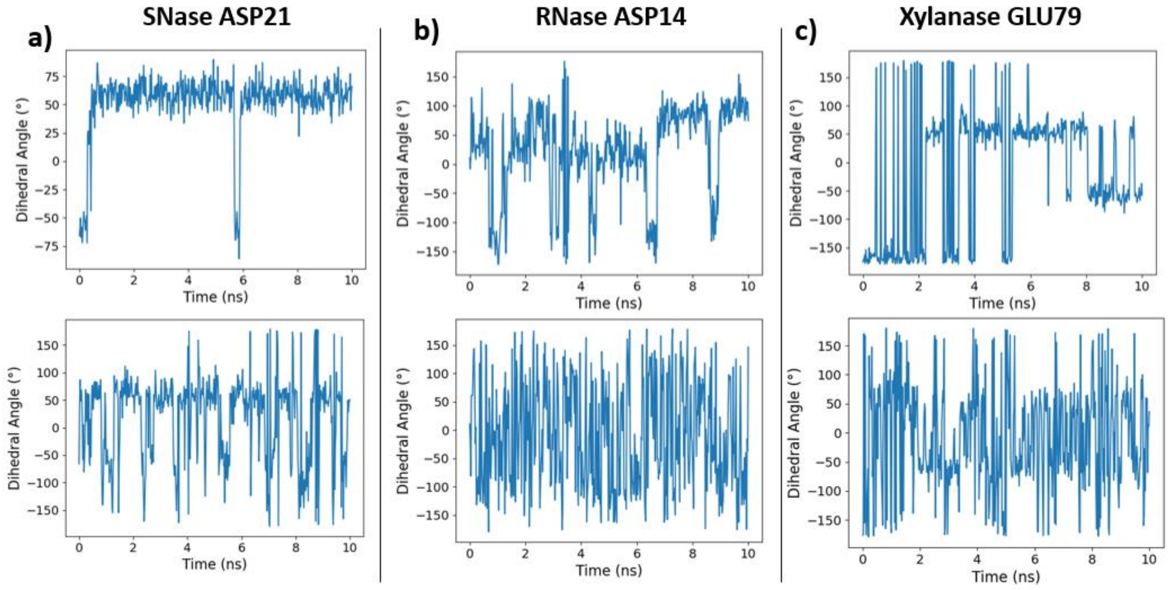

Figure S3. Trajectory torsion analysis for pH-AFED (top) and pH-AFED enhanced with dAFED (bottom) of a) ASP21 of SNase (N-C_γ-C_β-C_α), ASP14 of RNaseA (N-C_γ-C_β-C_α), and c) GLU79 of Xylanase ($O_{\delta 1}$-C_δ- C_β-C_α). The fluctuations observed in the top plot of c) for GLU79 from -180 to 180 are not due to large sudden rotations of the torsion angle, but from the periodicity in how the reference torsion angles are defined

Table S9. Comparison of pH-AFED performance with the reported results of other all-atom CpHMD implementations. The table reports the pKa RMSE values.

	**pH-AFED**	**pH AFED dAFED (10ns)**	**pH AFED dAFED (2 ns)**	**AMBER[4]**	**CHARMM[5]**
**HEWL**	0.68	0.76	0.48	0.83	0.92
**Thioredoxin**	0.44	0.36	0.39	0.71	-
**SNase**	0.98	0.89	1.05	0.76	0.8
**RNaseA**	1.01	0.84	1.10	0.81	-
**BBL**	0.64	0.49	0.69	0.62	0.66

a)

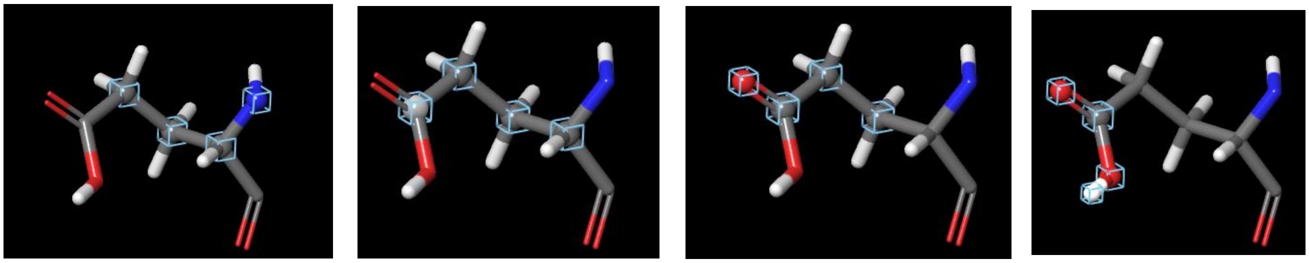

b)

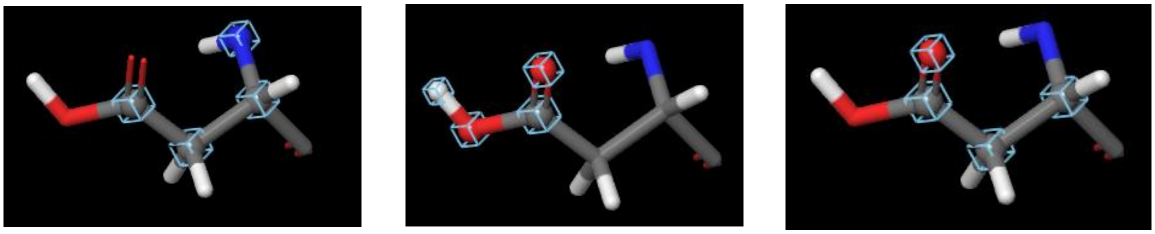

c)

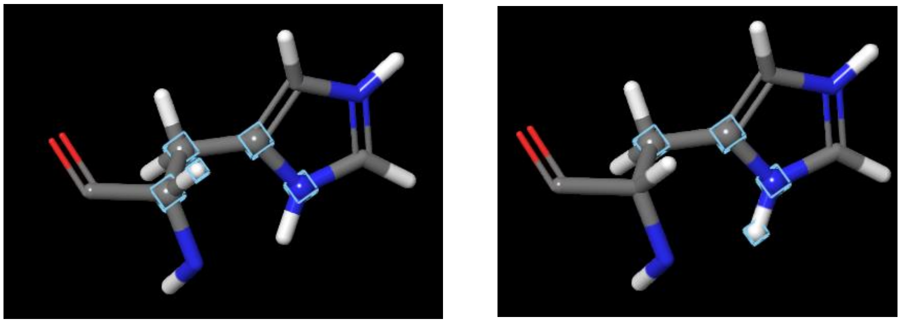

Figure S4. Depiction of torsion angles enhanced using d-AFED for a) GLU b) ASP c) HIS

References

1. Aho, N.; Buslaev, P.; Jansen, A.; Bauer, P.; Groenhof, G.; Hess, B., Scalable Constant pH Molecular Dynamics in GROMACS. *J. Chem. Theory Comput.* **2022,** *18* (10), 6148-6160.
2. Abrams, J. B.; Rosso, L.; Tuckerman, M. E., Efficient and precise solvation free energies via alchemical adiabatic molecular dynamics. *J Chem Phys* **2006,** *125* (7), 074115.
3. Rosso, L.; Mináry, P.; Zhu, Z.; Tuckerman, M. E., On the use of the adiabatic molecular dynamics technique in the calculation of free energy profiles. *J. Chem. Phys.* **2002,** *116* (11), 4389-4402.
4. Harris, J. A.; Liu, R.; Martins de Oliveira, V.; Vázquez-Montelongo, E. A.; Henderson, J. A.; Shen, J., GPU-Accelerated All-Atom Particle-Mesh Ewald Continuous Constant pH Molecular Dynamics in Amber. *J. Chem. Theory Comput.* **2022,** *18* (12), 7510-7527.
5. Huang, Y.; Chen, W.; Wallace, J. A.; Shen, J., All-Atom Continuous Constant pH Molecular Dynamics With Particle Mesh Ewald and Titratable Water. *J. Chem. Theory Comput.* **2016,** *12* (11), 5411-5421.
